# Multidisciplinary Management of Women Suffering from Migraine: Rationale, Design and Results of a National Delphi Consensus

**DOI:** 10.3390/healthcare14132014

**Published:** 2026-07-06

**Authors:** Piero Barbanti, Rossella E. Nappi, Sabina Cevoli, Patrizio Pasqualetti, Pasquale Perrone Filardi, Innocenzo Rainero, Alessandro Rossi, Vito Trojano, Annamaria Colao

**Affiliations:** 1 Headache and Pain Unit, IRCCS San Raffaele, 00163 Rome, Italy; piero.barbanti@sanraffaele.it; 2 Department of Neuroscience and Neurorehabilitation, San Raffaele University, 00166 Rome, Italy; 3 Department of Clinical, Surgical, Diagnostic and Pediatric Sciences, University of Pavia, 27100 Pavia, Italy; nappi@rossellanappi.com; 4 Research Center for Reproductive Medicine, Gynecological Endocrinology and Menopause, IRCCS S. Matteo Foundation, 27100 Pavia, Italy; 5 IRCCS Istituto Delle Scienze Neurologiche di Bologna, 40139 Bologna, Italy; sabina.cevoli@unibo.it; 6 Department of Wellbeing, Health & Environmental Sustainability, Sapienza University of Rome, 00185 Rome, Italy; 7 Department of Advanced Biomedical Sciences, University of Naples Federico II, 80131 Naples, Italy; pasquale.perrone@unina.it; 8 Center for Alzheimer’s Disease and Related Dementias, Department of Neuroscience and Mental Health, A.O.U. Città Della Salute e Della Scienza di Torino, 10126 Turin, Italy; innocenzo.rainero@unito.it; 9 Department of Neuroscience “Rita Levi Montalcini”, University of Torino, 10126 Turin, Italy; 10 Italian College of General Practitioners and Primary Care (SIMG), 50142 Florence, Italy; rossi.alessandro@simg.it; 11 Italian Society of Gynecology and Obstetrics (SIGO), 00187 Rome, Italy; vtrojano@katamail.com; 12 Endocrinology, Diabetology and Andrology Unit, Department of Clinical Medicine and Surgery, University of Naples Federico II, 80131 Naples, Italy; colao@unina.it; 13 UNESCO, Education for Health and Sustainable Development, University of Naples Federico II, 80131 Naples, Italy

**Keywords:** migraine in women, Delphi consensus, multidisciplinary care, menstrual migraine, sex-specific medicine

## Abstract

**Background/Objectives**: Migraine is a common, disabling neurological disorder disproportionately affecting women during hormonally sensitive phases like menarche, pregnancy, and menopause. Despite awareness of sex-specific risk factors, management remains fragmented and predominantly neurologist-led, with limited coordination. To address these gaps, this project aimed to develop a national expert consensus, endorsed by scientific societies, on multidisciplinary migraine management in women across the lifespan, integrating perspectives from neurology, gynecology, endocrinology, cardiology, and general medicine. **Methods**: A two-round Delphi survey was conducted among 145 Italian clinicians representing the five specialties. The Scientific Board formulated 50 questions, each consisting of a variable number of statements, covering diagnostic approaches, hormonal therapies, comorbidities, and organizational care pathways. Statements were rated on a nine-point Likert scale, and consensus was defined using pre-specified criteria based on median scores and agreement. **Results**: Overall, 79 of 145 statements (54%) achieved consensus. High-level agreement emerged on sex-informed diagnostic strategies, including systematic gynecological and endocrinological evaluation and hormonal profiling in women with migraine. Round 2 facilitated consensus on contentious issues, such as avoiding estrogen-containing contraceptives in migraine with aura, individualized thrombotic risk assessment during menopause, and structured interdisciplinary coordination—particularly among neurologists, gynecologists, and general practitioners—during fertility planning and assisted reproduction. Qualitative feedback emphasized the need to update clinical pathways, implement standardized referral models, and strengthen interprofessional communication. However, persistent divergence remained on selected topics, particularly the role of hormonal contraceptives as a first-line approach to migraine management in women of reproductive age, reflecting different priorities between gynecologists and neurologists. **Conclusions**: This Delphi initiative provides the first national multidisciplinary consensus on migraine management in women in Italy. The findings support the development of sex- and life-stage-specific clinical guidance and integrated care models tailored to the complex needs of women with migraine.

## 1. Introduction

Migraine is a highly prevalent and disabling neurological disorder that disproportionately affects women, especially during their reproductive years. Epidemiological data indicate that up to 18% of women experience migraine, with a peak incidence between the ages of 20 and 50 [1]. Hormonal fluctuations, especially those associated with menstruation, pregnancy, and menopause, are well-established modulators of migraine onset, frequency, and severity in women [2]. Beyond endocrine fluctuations, emerging evidence suggests that neuro-immune signaling pathways, including inflammatory mediators and oxytocin-related modulation, may contribute to sex-specific migraine expression and treatment response in women [3]. Furthermore, women with migraine frequently present with comorbidities such as cardiovascular risk factors, metabolic syndrome, endocrine disorders, and procoagulant states, all of which further complicate clinical management [4,5,6,7]. Endocrine and metabolic factors may further contribute to migraine susceptibility and chronicity in women. Hormonal fluctuations across the female lifespan, together with metabolic alterations frequently observed during reproductive and menopausal transitions, have been associated with changes in migraine burden and clinical expression [4,8]. Moreover, the hypothalamic–pituitary–gonadal axis plays a central role in the interaction between hormonal fluctuations and migraine expression across the female lifespan [8]. These considerations support the involvement of endocrinologists in the multidisciplinary assessment of women with migraine, particularly during hormonally sensitive phases and in the presence of metabolic comorbidities.

The interaction between hormonal status and migraine is particularly relevant when considering treatment options. Hormonal therapies, including combined hormonal contraception (CHC), menopausal hormone therapy (MHT) and other hormone-based treatments such as estradiol supplementation during the perimenstrual phase, may be appropriate in selected cases. However, these therapies, along with novel pharmacologic agents such as CGRP monoclonal antibodies, require individualized risk–benefit assessment, especially in women with vascular or thromboembolic risk factors [9,10]. Given that both migraine with aura and the administration of exogenous hormones can affect vascular risk, an individualized approach and interdisciplinary decision-making remain essential [11,12]. Moreover, emerging evidence has highlighted the potential impact of anti-CGRP therapies on bone metabolism, particularly in postmenopausal women or those receiving antiseizure medications [13], which further supports the need for a comprehensive, multidisciplinary evaluation.

Despite this clinical complexity, the management of migraine in women remains largely unidisciplinary and neurologist-led. Other specialists, such as gynecologists, endocrinologists, cardiologists, and general practitioners, are rarely involved in a structured or collaborative way, even though they play critical roles in addressing comorbidities and life-stage-specific challenges. This fragmented model may lead to underdiagnosis, therapeutic inertia, and suboptimal care, particularly in scenarios involving hormonal contraception, fertility treatments, periconceptional period, menopausal transition, or cardiovascular and thrombotic risk stratification [8,14].

In response to this unmet need, recent expert recommendations and global health strategies have emphasized the importance of adopting sex- and gender-informed, multidisciplinary approaches for chronic conditions affecting women, including migraine [15,16]. Nevertheless, no Delphi consensus studies to date have systematically explored cross-specialty agreement on how best to manage migraine in women through an integrated clinical framework.

To address this gap, a nationwide Delphi consensus project was initiated in Italy. The aim was to develop a multidisciplinary, evidence-informed study on the diagnosis, treatment, and care coordination of migraine in women, involving five key specialties: neurology, gynecology, endocrinology, cardiology, and general medicine. The project was led by national Scientific Societies and coordinated by a Scientific Board composed of eight Key Opinion Leaders with apical roles in their respective scientific societies and an Expert Panel of 145 Italian clinicians. The process was structured in two iterative rounds, which explored both general and life-stage-specific aspects of migraine care, including adolescence, migraine in the fertile period, pregnancy, lactation, menopause, and post-menopause.

The insights generated through this initiative are expected to inform future clinical recommendations and improve the quality, safety, and integration of migraine care for women across their lifespan.

## 2. Materials and Methods

### 2.1. Study Design

This study employed a two-round Delphi methodology to develop expert consensus on the multidisciplinary management of migraine in women across different life stages. The Delphi method-characterized by anonymity, controlled feedback, and iterative consensus-building-is widely recognized in health research for synthesizing expert opinion [17,18,19,20,21].

The initiative was conducted nationally in Italy and aimed to capture both converging and diverging views among five key specialties involved in the management of migraine and its comorbidities: neurology, gynecology, endocrinology, cardiology, and general medicine. A Scientific Board composed of members of Scientific Societies with at least one representative from each specialty-oversaw study design, questionnaire development, and interpretation of results.

### 2.2. Participants and Recruitment

Expert panelists were recruited via national scientific societies and specialty-specific professional networks. Eligible participants were Italian clinicians with active clinical roles in one of the five target specialties who were routinely involved in the management of women with migraine or migraine-related comorbidities within their area of practice. To characterize the expertise of the panel, information on clinical setting, years of professional experience, and annual volume of migraine patients was collected.

All participants received background literature and were invited to complete both Delphi rounds via a secure online platform. Participation was voluntary, and informed digital consent was obtained. The final panel included 145 clinicians, distributed across the five specialties.

### 2.3. Delphi Procedure

In Round 1, participants evaluated 34 questionnaire items, including 31 statements rated on a 9-point Likert scale (1 = strongly disagree; 9 = strongly agree) and 3 ranking questions in which response options were ordered according to perceived importance.

Thematic areas included:

1. MIGRAINE AND PUBERTY
2. MIGRAINE IN THE FERTILE PERIOD
3. MIGRAINE AND HORMONAL CONTRACEPTION/TREATMENTS
4. MIGRAINE IN PREGNANCY AND BREASTFEEDING
5. MIGRAINE IN MENOPAUSE AND POST-MENOPAUSE
6. MIGRAINE DIAGNOSIS AND STIGMATIZATION

Items that did not meet the predefined consensus threshold or received substantial feedback were revised by the Scientific Board. Round 2 included reformulated items and new statements addressing specific clinical scenarios across the female life cycle—such as adolescence, migraine in the fertile period, pregnancy, breastfeeding, menopause, and postmenopause. All items in both rounds were evaluated using the same Likert scale (Supplementary Material-Table S7), except for four questions using alternative response formats (multiple-choice or ranking) that were included to capture panelists’ perspectives on selected organizational or clinical aspects (Supplementary Material-Figure S1).

The entire process was managed online, ensuring anonymity, national reach, and iterative reflection. Quantitative and qualitative feedback were analyzed after each round to finalize consensus outcomes.

### 2.4. Data Collection and Analysis

Demographic data (specialty, region of practice) were collected to enable subgroup analysis. Individual responses were anonymized prior to processing. Survey data were collected using an encrypted digital platform and analyzed in aggregate form.

Quantitative agreement levels were calculated for each item, both overall and by specialty subgroup. Respondents’ agreement was assessed using the Delphi method, following the guidelines established by the RAND Corporation [22]. This method utilizes a scale ranging from 1 (maximum disagreement) to 9 (maximum agreement), with a score of 5 representing a neutral opinion on a specific item. The scores provided by respondents were then statistically analyzed to calculate an appropriate “consensus index”. In line with “The RAND/UCLA Appropriateness Method User’s Manual”, the Inter-Percentile Range Adjusted for Symmetry (IPRAS) was used as a measure of score dispersion adjusted for symmetry, to determine the level of agreement for each item. The rationale is that when ratings are symmetrical, the Inter-Percentile Range (IPR) required to classify an indication as disagreement is smaller compared to when the ratings are asymmetrical. Asymmetry was defined as “the distance between the central point of the IPR and the central point of the 1–9 scale, i.e., 5”. As the asymmetry of ratings increases, a larger IPR is required to indicate disagreement. The following mathematical function was developed: IPRAS = IPRr + (AI × CFA), where IPRr is the IPR required for disagreement under perfect symmetry, AI is the Asymmetry Index, and CFA is the Correction Factor for Asymmetry. The IPRAS threshold is dependent on the symmetry of the ratings around the median, meaning that each item requires its own IPRAS calculation. Consequently, an item is rated as having disagreement if IPRi > IPRAS. Based on the computation of IPR and IPRAS, consensus was assessed according to the RAND/UCLA Appropriateness Methos. Statements with a panel median of 7–9 and no disagreement (IPRi > IPRAS) were classified as “Appropriate”, whereas statements with a median of 1–3, without disagreement, were classified as “Inappropriate”. Statements with a median of 4–6, or any statement showing disagreement, were classified as “Uncertain”.

Data analysis was performed using SPSS 29.0 (IBM), and a scoring sheet was developed in Excel to calculate all necessary statistics as outlined in “The RAND/UCLA Appropriateness Method User’s Manual”.

### 2.5. Ethical Considerations

The study complied with current EU and Italian data protection and research ethics regulations (GDPR-compliant). All participants provided digital informed consent. As the study involved only healthcare professionals, did not collect any sensitive personal data, and did not involve patients or interventions, submission to an ethics committee was not required under current Italian regulations governing expert consensus research.

## 3. Results

A total of 145 Italian clinicians participated in the Delphi survey, differently representing five core specialties: 60 neurology, 32 gynecology, 26 endocrinology, 14 cardiology, and 13 general medicine (Table 1). Respondents formed an experienced cohort, with a mean age of 50.7 years (SD = 11.9) and an average of 25.1 years since graduation (SD = 12.2). Most practiced in public hospitals (55%) or university-affiliated centers (30%), while others worked in private or community outpatient settings.

Clinicians reported different volumes of migraine cases according to their specialties, with 93% of neurologists indicating more than 100 migraine cases in a year while the majority of the other specialists see less than 50 cases/year. On the other hand, the prevalence of women among migraine patients was quite homogenous, reaching at least 75% for every specialty. Most female migraine patients were aged 19–50, although a relevant proportion were perimenopausal or postmenopausal (Supplementary Material-Table S1). These age-related differences highlight the importance of endocrine assessment, particularly in perimenopausal and postmenopausal women, in whom hormonal changes, metabolic risk factors, and bone health may influence both migraine expression and therapeutic decisions.

Despite this, while most respondents reported the presence of a dedicated migraine treatment center within their facility (e.g., 86% of cardiologists, 96% of endocrinologists), only a minority indicated the existence of a formal care pathway (PDTA) for migraine management. Interspecialty collaboration was often informal or absent, and referral models involving general practitioners remained largely unstructured.

**Figure 1:**
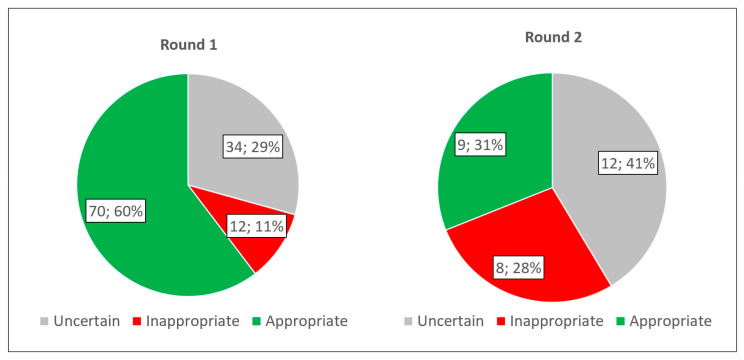
Number and percentage of consensus responses reached for the items in each round.

**Figure 2:**
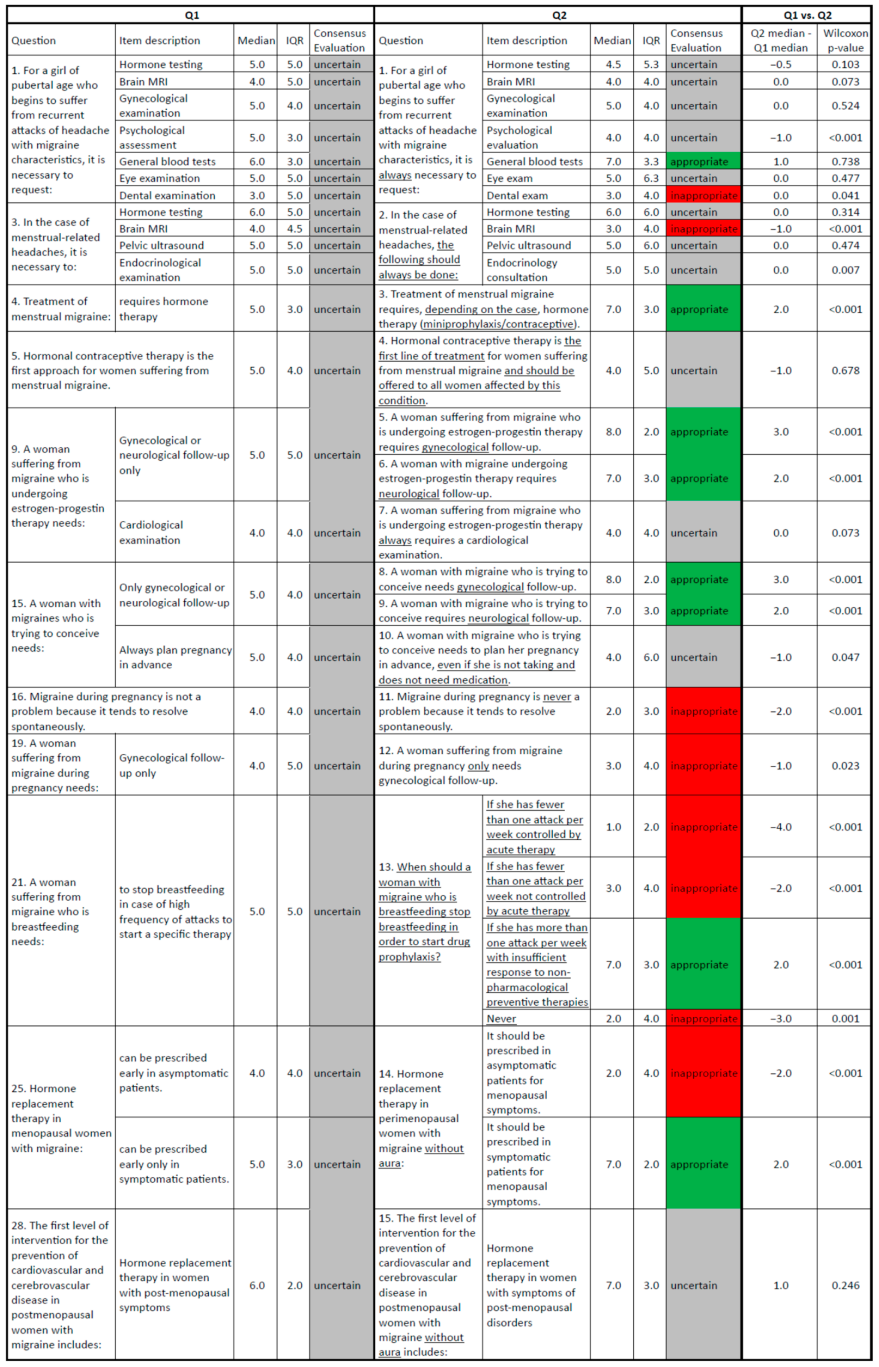
Delphi results: Comparison Q1 vs. Q2 results. The underlined texts indicate the new wording of the question in Round 2.

The Delphi process was conducted in two iterative rounds. In Round 1, 34 questions, many of which consist of a variable number of statements, were assessed across four thematic domains: diagnosis, hormonal therapies/treatments, comorbidities, and multidisciplinary management. Consensus, defined as negative agreement for ratings of 1 to 3 or positive agreement for ratings of 7 to 9, was achieved for 70 out of 116 statements (60%). Based on participant feedback, 11 of the original questions were revised, and 5 new ones were added in Round 2, resulting in 16 questions that explored specific clinical scenarios across the female lifespan, including adolescence, migraine in the fertile period pregnancy, breastfeeding, menopause, and assisted reproduction. In Round 2, 9 statements reached consensus, raising the total to 79 out of 145 (54%) across both rounds (Figure 1). Notably, the statements that reached consensus in Round 2 had failed to achieve agreement in Round 1, underscoring the value of iterative refinement in resolving uncertainty and aligning cross-specialty perspectives (Figure 2). Overall, consensus was most frequently achieved for sex-informed diagnostic approaches, reproductive history assessment, interdisciplinary referral pathways, and migraine management across key hormonal transitions. Conversely, lower levels of agreement were observed for selected aspects of hormonal therapy, thrombophilia screening, pharmacologic treatment during breastfeeding, and the role of general practitioners in migraine care.

Thematic analysis revealed strong alignment in diagnostic approaches incorporating hormonal profiling, gynecologic history, and sex-specific criteria. In particular, systematic hormonal and metabolic assessment—areas in which endocrinological evaluation may contribute to a more comprehensive characterization of female migraine patients—were widely recognized as relevant components of the diagnostic workup. Broad agreement also emerged across specialties on the routine inclusion of contraceptive use assessment in women presenting with migraine.

Round 2 enabled new consensus on several previously divisive items. For example, the panel moved from uncertainty to a consensus judgment of inappropriateness on the claim that ‘migraine is never a problem during pregnancy as it tends to resolve spontaneously,’ thereby countering a clinical misconception that could contribute to undertreatment. Regarding breastfeeding, consensus was achieved on a pragmatic threshold for pharmacologic prophylaxis, endorsing the interruption of breastfeeding only in cases where the patient experiences more than one attack per week with insufficient response to non-pharmacological therapies. These statements helped establish clearer criteria for clinical decision-making in hormonally sensitive phases.

Among the other items revised in Round 2, an agreement was reached on:

- Avoidance of estrogen-containing contraceptives in women with migraine with aura.
- The need for cardiovascular risk assessment and individualized therapeutic strategies in women with migraine and vascular risk factors.
- Recognition of migraine as a comorbidity during contraceptive, fertility, and menopause counseling.
- Integration of neurologists, gynecologists, and general practitioners in fertility and ART care; the consensus highlights the importance of multidisciplinary coordination in assisted reproduction settings, suggesting that neurological clearance should be considered in women with migraine history before initiating IVF, particularly in the presence of aura or vascular risk factors.

In addition, the panel clearly rejected the appropriateness of routine dental examinations for the diagnosis of migraine in pubertal girls, underscoring the need to avoid unnecessary or irrelevant referrals. Conversely, there was uncertainty regarding the role of eye examinations-often requested by general practitioners-as a diagnostic step. This likely reflects variability in practice, and points to the need for clearer referral algorithms that prevent redundant testing while ensuring comprehensive evaluation.

Among the 145 statements submitted to expert evaluation across the two Delphi rounds, several reached immediate and strong consensus in Round 1, highlighting robust cross-specialty agreement on fundamental clinical principles. Four questions were included using alternative response formats (one multiple-choice item and three ranking questions). Although these items were not rated using the 9-point Likert scale and have not reached full agreement, they provide a descriptive overview of panelists’ opinions and offer contextual insights into selected aspects of clinical practice (Supplementary Material-Figure S1). Notably, full consensus emerged on the relevance of sex-informed approaches to diagnosis, including the integration of family history, gynecologic history and contraception, elements endorsed as “appropriate” by all specialties (Supplementary Material-Table S2, question 2-item 6, question 3-item 6; Table S5, item 30). This convergence reflects a growing clinical awareness that hormonal fluctuations and reproductive milestones significantly influence migraine patterns, severity, and therapeutic response in women. In selected cases, endocrine evaluation—including assessment of thyroid function, metabolic parameters, and reproductive hormones—may support a more comprehensive diagnostic workup. Endorsing these elements as essential components of diagnostic assessment underscores the importance of personalized, sex-specific evaluation in improving accuracy, treatment planning, and patient outcomes. Similarly, high agreement was found regarding the prioritization of preventive goals in migraine management (e.g., reduction in attacks, complications, and disability), and the need to address stigma and misrecognition in women affected by migraine (Supplementary Material-Table S6, question 31, 33). These findings reinforce the recognition of migraine as a chronic and potentially disabling condition in women, requiring proactive and individualized long-term strategies. Addressing stigma and ensuring appropriate recognition in clinical settings may improve adherence, reduce diagnostic delays, and support comprehensive care planning, especially during hormonally sensitive periods.

Conversely, some items initially failed to achieve consensus and required significant revision in Round 2. In particular, items addressing thrombotic risk stratification, hormonal contraception in women with migraine with aura, and cardiovascular screening in perimenopausal patients-initially met with uncertainty or divergence-achieved consensus only after being clarified or reframed (Figure 2, items 7, 8, 25, 26). This evolution illustrates how iterative reflection contributed to aligning perspectives on complex, risk-sensitive scenarios. Clinically, these areas are pivotal for patient safety, as they intersect with known vascular vulnerabilities in women with migraine, notably those with aura. The emergence of consensus supports the adoption of more cautious and individualized approaches to hormonal and cardiovascular management in these patients and provides a clearer framework for interdisciplinary risk assessment in everyday practice.

Nonetheless, persistent divergence remained on a small number of items. For example, marked differences emerged between gynecologists and neurologists regarding the appropriateness of hormonal contraceptive therapy as the first-line approach for migraine in the fertile period. While gynecologists tended to consider it appropriate due to its role in hormonal stabilization, neurologists often deemed it inappropriate, favoring specific migraine preventive agents instead. This reflects the distinct clinical priorities and interpretative frameworks of each specialty, highlighting the ongoing need for interdisciplinary dialog and shared decision-making in managing hormonally mediated migraine. Notably, systematic thrombophilia screening in women with migraine, the use of pharmacologic therapy during breastfeeding, and the structured role of general practitioners in migraine care continued to show heterogeneous responses even after revision and re-evaluation (Supplementary Material-Table S2, question 4, 5; Table S3, question 9; Table S4, question 21; Figure 2).

A summary of the key clinical suggestions emerging from this Delphi process is provided in Table 2, organized by diagnostic, therapeutic, and organizational domains. The complete list of all statements with corresponding agreement levels across both rounds is reported in the Supplementary Material-Table S7.

## 4. Discussion

This Delphi consensus study represents the first structured, multidisciplinary effort in Italy to define shared clinical principles for the management of migraine in women. The high participation rate and balanced representation of five key specialties reflect a strong interest in improving care pathways for a patient population often affected by fragmented management and underrecognized clinical complexity.

The high consensus rates observed in the domains of diagnosis and multidisciplinary management suggest a growing awareness among clinicians of the need to adopt sex-specific approaches and interprofessional collaboration. In particular, Round 1 revealed immediate and unanimous consensus on core diagnostic practices, such as the integration of reproductive history, gynecologic evaluation, and hormonal profiling, highlighting robust alignment across specialties on foundational principles. All disciplines strongly endorsed the importance of systematically assessing gynecologic and contraceptive history, especially in the context of menstrual-related headaches, underscoring the need to incorporate these factors into routine migraine evaluation in women. Conversely, general practitioners expressed uncertainty regarding the appropriateness of certain diagnostic investigations, such as brain MRI or hormonal assays in pubertal girls with headache. This divergence highlights the need for clearer referral protocols and interspecialty collaboration to ensure appropriate use of healthcare resources and avoid unnecessary testing.

Furthermore, consensus emerged on the importance of active preconception planning and follow-up for women with migraine who are trying to conceive. Respondents agreed that these patients require coordinated care involving both neurologists and gynecologists to optimize treatment decisions and reduce risk exposure, particularly in cases of migraine with aura or comorbid conditions.

Conversely, lower consensus levels in the areas of hormonal therapy and comorbidities highlight the persistence of clinical uncertainty and variability in practice. Endocrine and cardiovascular comorbidities, including overweight or obesity, insulin resistance, thyroid disorders, dyslipidemia, hypertension and menopausal hormonal changes, may significantly modulate migraine burden and therapeutic choices, highlighting the potential contribution of endocrinologists and cardiologist within multidisciplinary care pathways. In addition to endocrine and vascular factors, psychiatric comorbidities, particularly depression, also play a major role in shaping disease burden and therapeutic outcomes in women with migraine. For instance, neurologists expressed skepticism regarding the routine use of hormonal screening in pubertal girls with migraine, reflecting concerns over the potential for unnecessary laboratory investigations in young patients without clear clinical indications. This divergence suggests the need for more nuanced, symptom-driven diagnostic protocols in adolescent populations.

Additionally, the role of BMI as a clinical parameter elicited mixed responses, notably in relation to migraine comorbidities. Endocrinologists emphasized the value of BMI assessment in identifying metabolic risk factors and tailoring hormonal or pharmacologic therapies, whereas other specialties were less consistent in recognizing its relevance, pointing to a potential gap in the integration of endocrine perspectives into routine migraine care. The management of hormonal contraception, menopausal hormone therapy (MHT), and reproductive counseling in women with migraine, especially those with aura or vascular risk factors, remains an area of controversy across specialties. In particular, Round 2 clarified that MHT is considered inappropriate for asymptomatic women with migraine, but appropriate in symptomatic women without aura, reflecting an individualized, symptom-driven approach to menopausal care. This distinction underscores the importance of tailoring hormonal interventions based not only on migraine phenotype but also on the severity of menopausal symptoms and overall vascular and metabolic risk. These findings mirror limited quality of the available evidence in the literature, underlining the need for individualized risk assessment and interdisciplinary decision-making [9,10,12].

An additional area requiring deeper multidisciplinary discussion is the potential impact of novel pharmacologic agents, such as CGRP monoclonal antibodies, on bone metabolism, notably in postmenopausal women or those receiving enzyme-inducing antiseizure medications. While these therapies offer a valuable alternative for migraine prevention, their long-term safety profile in women at risk of osteoporosis remains insufficiently explored [13]. In this context, endocrinologists may play a key role in the long-term follow-up of patients receiving anti-CGRP therapies, particularly in postmenopausal women or in those exposed to antiseizure medications, by monitoring bone health and helping ensure the skeletal safety of patients with chronic migraine. Experimental data suggest that CGRP is also involved in immune regulation and bone homeostasis, indicating that long-term blockade may warrant careful endocrine monitoring in selected populations [23]. Future guidance should explicitly address this issue, promoting shared decision-making protocols that integrate hormonal, neurologic, and metabolic risk factors.

Notably, the second Delphi round enabled significant convergence on previously contentious issues. An agreement was achieved on key aspects of cardiovascular risk stratification, contraceptive counseling in women with migraine with aura, and therapeutic decision-making in complex clinical scenarios. These findings reflect a clinically meaningful shift toward more structured, risk-informed, and individualized management approaches, particularly in patients with comorbidities or contraindications to hormonal strategies. This evolving confidence is paralleled by the rapid expansion of the global clinical trial landscape investigating CGRP-targeted therapeutics, reflecting a sustained translational momentum from pathophysiological discovery to preventive treatment development [24]. This endorsement may guide safer, individualized treatment pathways for women at increased vascular risk. After targeted refinement, several items addressing thrombotic risk stratification, contraceptive counseling in women with aura, and cardiovascular screening during perimenopause reached the predefined threshold for consensus (Figure 2). In total, 9 of the 29 items submitted in Round 2 achieved consensus, contributing to a final total of 79 out of 145 consensus statements (54%). Persistent divergence remained, however, on a few sensitive topics, such as systematic thrombophilia screening, pharmacologic treatment during breastfeeding, and the structured role of general practitioners-suggesting areas that may require further clarification through research, training, or specialty-specific guidelines [25,26,27].

This Delphi process highlighted both areas of strong convergence and domains of persistent divergence. Immediate cross-specialty agreement on diagnostic practices, such as reproductive history, gynecologic evaluation and hormonal profiling reflects a broad readiness to adopt sex- and life-stage-informed approaches. Similarly, there was robust consensus on prioritizing preventive goals and addressing stigma in women.

In contrast, topics such as hormonal contraception in migraine with aura, thrombotic risk assessment, and menopause or ART-related care initially drew mixed responses. One notable example of persistent divergence concerned the use of combined hormonal contraceptives as a first-line approach to migraine management in women of reproductive age. This difference likely reflects distinct risk–benefit perspectives across specialties. Neurologists may be more cautious because of the well-established association between migraine with aura and ischemic stroke risk, particularly when aura symptoms are underrecognized or inconsistently reported. In contrast, gynecologists often prioritize hormonal stabilization and cycle regulation, which may help reduce the frequency and predictability of menstrual-related migraine attacks. These differing perspectives underscore the importance of individualized assessment and multidisciplinary decision-making when selecting therapeutic strategies for women with hormonally related migraine [2,12]. These areas were progressively refined through iterative dialog, leading to new consensus in Round 2. However, some issues—like systematic thrombophilia screening, pharmacologic treatment during breastfeeding, and the role of general practitioners—remained divisive, reflecting both clinical complexity and heterogeneous practices. The overall consensus rate of 58% may appear modest compared with that reported in some Delphi studies; however, this finding likely reflects the complexity of the topics addressed and the genuinely multidisciplinary composition of the panel. By design, the study brought together specialists with different clinical priorities and decision-making frameworks across neurological, gynecological, endocrine, cardiovascular, and primary care settings. Consequently, areas characterized by limited evidence, evolving recommendations, or competing clinical considerations were expected to generate greater variability in responses. In this context, persistent disagreement should be viewed not only as a lack of consensus but also as an indicator of topics requiring further research, education, and interdisciplinary dialogue.

These findings carry important implications for clinical practice. Strong agreement supports the development of shared care pathways and referral models tailored to women’s hormonal life stages. Conversely, unresolved items point to priority areas for further research, continuing education, and specialty-specific guidance.

Our findings are consistent with prior national studies highlighting the organizational fragmentation and limited integration of care pathways for women with migraine. Additional exploratory questions also underscored the perceived relevance of endocrine and metabolic aspects in the management of female migraine, reinforcing the need for multidisciplinary collaboration (Supplementary Material-Figure S1). The Delphi panel identified specific operational priorities to enhance care coordination, including interdisciplinary referral pathways involving neurologists, gynecologists, and general practitioners during key hormonal transitions such as pregnancy planning, menopause, and assisted reproduction. Consensus also emerged on the need for structured clinical pathways (PDTA), shared documentation tools, and coordinated follow-up strategies to improve continuity of care. Together, these findings provide a practical framework for the development of multidisciplinary, life-course-oriented clinical guidance for women with migraine. A recent Italian analysis underscored the absence of PDTA and the underutilization of general practitioners in the diagnosis and management of migraine [16]. The findings of our Delphi study offer preliminary criteria to support clearer delineation of roles between general practitioners and specialist care. For instance, consensus emerged that GPs may manage patients with infrequent or stable migraine without red flags; however, referral to a headache center or neurologist is appropriate in cases of diagnostic uncertainty, disabling attack frequency (e.g., more than 4–5 per month), or when initiating preventive treatments. Importantly, active referral was also endorsed for hormonally sensitive phases such as pregnancy planning, perimenopause, or when migraine presents with aura or vascular comorbidities. These criteria may serve as the foundation for future shared care agreements within regional healthcare systems. From a practical perspective, the findings support adapting existing care pathways to include structured multidisciplinary referral points during key hormonal transitions, such as pregnancy planning, assisted reproduction, and menopause. Standardized referral criteria based on migraine frequency, aura status, vascular risk factors, and reproductive plans may facilitate timely escalation from primary care to specialist services. Rather than replacing existing algorithms, these recommendations integrate sex- and life-stage-specific decision points to promote coordinated care. These structural gaps may lead to care delays, suboptimal treatment decisions, and insufficient attention to sex-specific risk factors. In this context, the high level of consensus reached in our study regarding the need for interdisciplinary collaboration and referral models further confirms the urgency of developing shared care frameworks, such as structured diagnostic and therapeutic pathways (PDTA), standardized referral protocols between general practitioners and specialists, and coordinated follow-up plans during key hormonal transitions (e.g., pregnancy, menopause, ART), especially within regional and national health systems.

Recent international studies reinforce the need for a multidimensional approach to female migraine care [28,29]. Emerging translational research has also highlighted the interaction between hormonal signaling, vascular function, and inflammatory pathways in migraine pathophysiology, further supporting the clinical relevance of interdisciplinary collaboration [30]. A recent review provides a mechanistic overview of migraine in the fertile and non-fertile period, highlighting the interplay between hormonal fluctuations, circadian rhythm disturbances, and gut–brain axis dysfunction [29]. Similarly, expert consensus has emphasized the underrecognition of fertile period phenotypes and the value of standardized hormonal history-taking during clinical assessment-an approach that closely mirrors the high-consensus diagnostic recommendations in our Delphi findings [29].

From a methodological perspective, the Delphi technique proved effective in eliciting expert agreement while preserving anonymity and independence. The iterative design allowed for the refinement of statements and consensus-building, while the inclusion of free-text feedback provided qualitative depth and contextual nuance. Notably, several clinicians used these open fields to raise real-world concerns regarding adverse events, off-label uses, or safety monitoring, underscoring the potential of free-text input as a complementary tool for pharmacovigilance and clinical risk identification in consensus-based research. The use of a multidisciplinary panel ensured that both convergent and divergent perspectives were captured-an essential condition for developing comprehensive and inclusive care models.

### Strengths and Limitations

This study presents several methodological strengths. First, the use of a two-round Delphi design enabled structured consensus-building while allowing for reflection and iterative refinement of statements. The anonymous and asynchronous format facilitated honest feedback and minimized group pressure, which is particularly valuable in multidisciplinary panels with potentially divergent perspectives.

Second, the panel composition ensured balanced representation from five core medical specialties—neurology, gynecology, endocrinology, cardiology, and general medicine—providing a comprehensive view of clinical practice across the female lifespan. Notably, the inclusion of endocrinologists allowed the panel to fully consider the endocrine dimensions of migraine, supporting a more nuanced understanding of hormonal influences across the female lifespan. The engagement of 145 experienced clinicians from diverse care settings further enhanced the validity and generalizability of the findings within the Italian healthcare context.

Third, the inclusion of both quantitative agreement rates and qualitative feedback allowed for a more nuanced interpretation of consensus levels. The Scientific Board’s active role in reviewing and refining items between rounds was instrumental in addressing ambiguities and ensuring thematic relevance.

In addition, the study was funded by a sponsor; however, the sponsor played no role whatsoever in formulating the statements, analyzing, or interpreting the results. However, some limitations must be acknowledged. The findings reflect expert opinion and are not a substitute for empirical outcome data. Although the panel was diverse, participation was voluntary and may have introduced selection bias, favoring clinicians with a preexisting interest in migraine or women’s health. Moreover, some items failed to reach consensus due to persistent interspecialty variability, which may limit the immediate applicability of certain recommendations.

Although the multidisciplinary composition of the panel represents a major strength, the larger proportion of neurologists compared with other specialties may have influenced overall agreement rates and should be considered when interpreting the results. Furthermore, the findings should be interpreted within the context of the Italian healthcare system and referral pathways, which may limit their direct generalizability to other healthcare settings. Finally, substantial differences in migraine-related clinical exposure were observed across specialties, with neurologists reporting considerably higher patient volumes than other panelists. This disparity may have influenced the relative weight of clinical experience in shaping some consensus positions. In addition, formal statistical comparisons between specialty groups were not performed, as the primary aim of the Delphi process was to assess consensus within a multidisciplinary panel rather than to test differences between specialties.

## 5. Conclusions

This multidisciplinary Delphi consensus represents a key step toward developing shared, sex-specific clinical guidance for the management of migraine in women. By integrating perspectives from five specialties, the study highlights a strong readiness for multidisciplinary, life-stage-informed care, while also identifying areas of persistent clinical uncertainty. The final set of 29 consensus statements provides a solid foundation for improving diagnostic approaches, therapeutic decision-making, and care coordination. Future efforts should focus on translating these insights into structured clinical pathways and integrated care models to enhance the quality and continuity of migraine care for women across their lifespan.

## Figures and Tables

**Table 1:** Distribution by specialty of the clinicians who participated in the two rounds of the Delphi Study.

Specialty	Round 1		Round 2	
	*n*	%	*n*	%
Cardiology	14	10%	9	8%
Endocrinology	26	18%	18	15%
Gynecology	32	22%	26	22%
General medicine	13	9%	13	11%
Neurology	60	41%	53	45%
Total	145	100%	119	100%

**Table 2:** Summary of key suggestions emerging from the Delphi Consensus.

Area	Suggestions	Consensus Strength
Diagnosis and Clinical Characterization	Integrate gynecologic and hormonal history into migraine diagnosis	Strong (Round 1)
	Use sex-specific diagnostic frameworks	Strong (Round 1)
Hormonal Therapies	Avoid estrogen-containing contraceptives in women with aura	Achieved in Round 2
	Consider individualized therapeutic strategies in patients with vascular risk factors	Achieved in Round 2
Comorbidities	Recognize migraine as a relevant comorbidity in contraceptive, ART and menopause counseling	Achieved in Round 2
	Stratify cardiovascular risks	Achieved in Round 2
Multidisciplinary Care and Referral	Establish interdisciplinary care pathways	Strong (Round 1)
	Involve gynecologists, general practitioners, endocrinologists, and cardiologists in complex cases	Achieved in Round 2

## Data Availability

The data that support the findings of this study are available from the corresponding author, upon reasonable request.

## Supplementary Materials

The following supporting information can be downloaded at: https://www.mdpi.com/article/10.3390/healthcare14132014/s1, Figure S1: Round 1 and Round 2 questions using alternative response formats; Table S1: Migraine management distribution; Table S2: Consensus Q1–Topic PUBERTY AND MENSTRUAL; Table S3: Consensus Q1–Topic HORMONAL CONTRACEPTION; Table S4: Consensus Q1–Topic PREGNANCY AND BREASTFEEDING; Table S5: Consensus Q1–Topic MENOPAUSE; Table S6: Consensus Q1–Topic DIAGNOSIS AND STIGMATIZATION; Table S7: All the statements with corresponding agreement levels across both rounds of the Delphi process; Supplementary Material–Participants.
