# Supplementary material for: Multidisciplinary Management of Women Suffering from Migraine: Rationale, Design and Results of a National Delphi Consensus
Source: Healthcare (Basel). 2026 Jul 6;14(13):2014. doi: 10.3390/healthcare14132014 (PMC13361374; doi:10.3390/healthcare14132014)

Supplementary Material - Figure S1. Round 1 and Round 2 questions using alternative response formats

A

Round 1 – Question 14. A patent foramen ovale (PFO) examination in women with migraine is indicated:

		Frequency	Valid Percent
Valid	1 Always	25	17,7
	2 Only in cases of aura	25	17,7
	3 Only in cases of aura with clear, documented cerebral ischemic lesions	78	55,3
	4 Not relevant	13	9,2
	Total	141	100,0
Missing	System	4	
Total		145	

B

Round 1 – Question 26. The following symptom/sign should be identified in women with migraine during peri- and post-menopause in relation to cardiovascular risk:

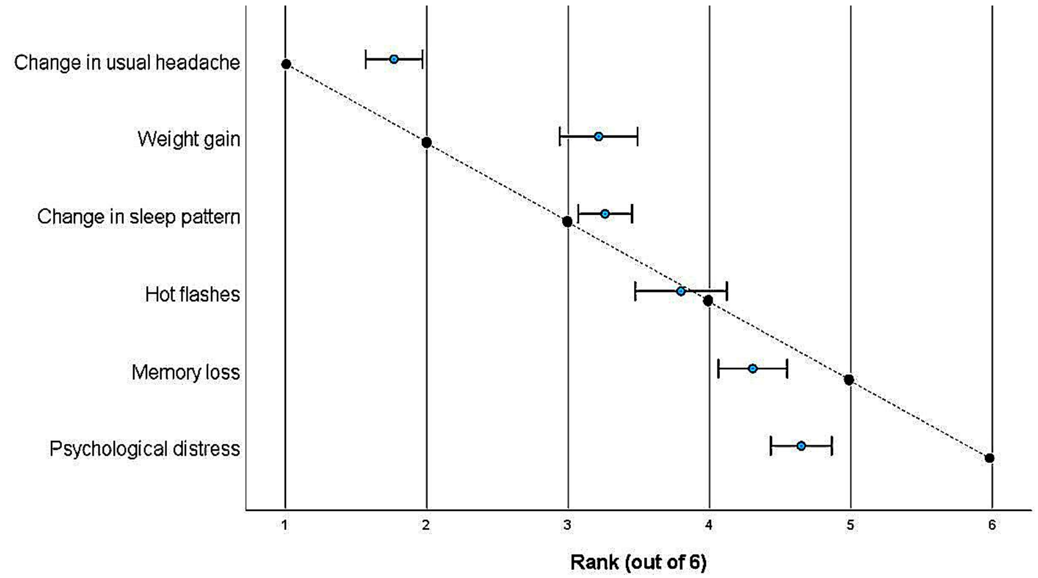

C

Round 1 – Question 27. For a patient with migraine in peri- and post-menopause with cardiovascular risk factors, I consider consultation necessary:

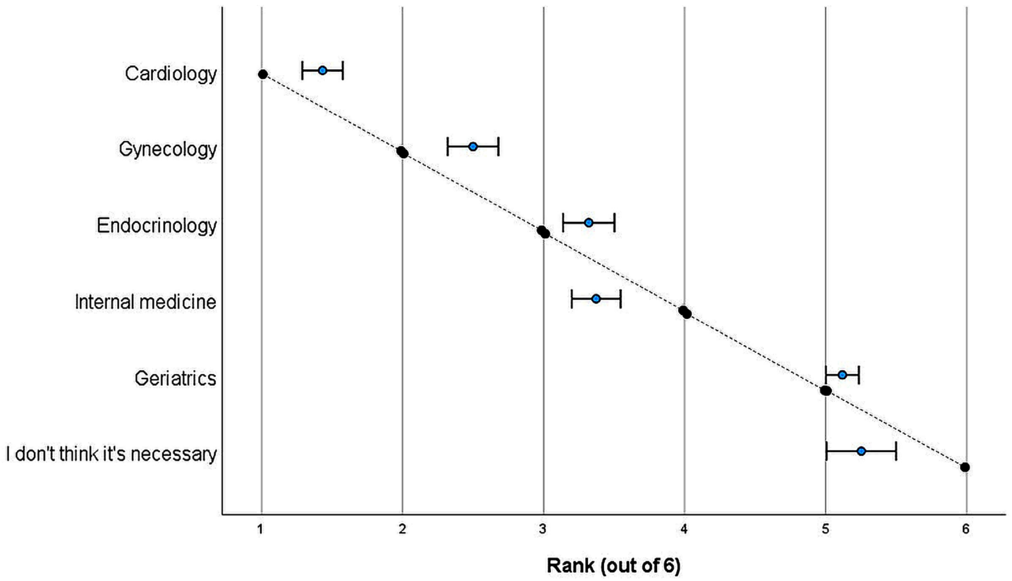

D

Round 2 – Question 16. Which healthcare professional should a woman with migraine consult?

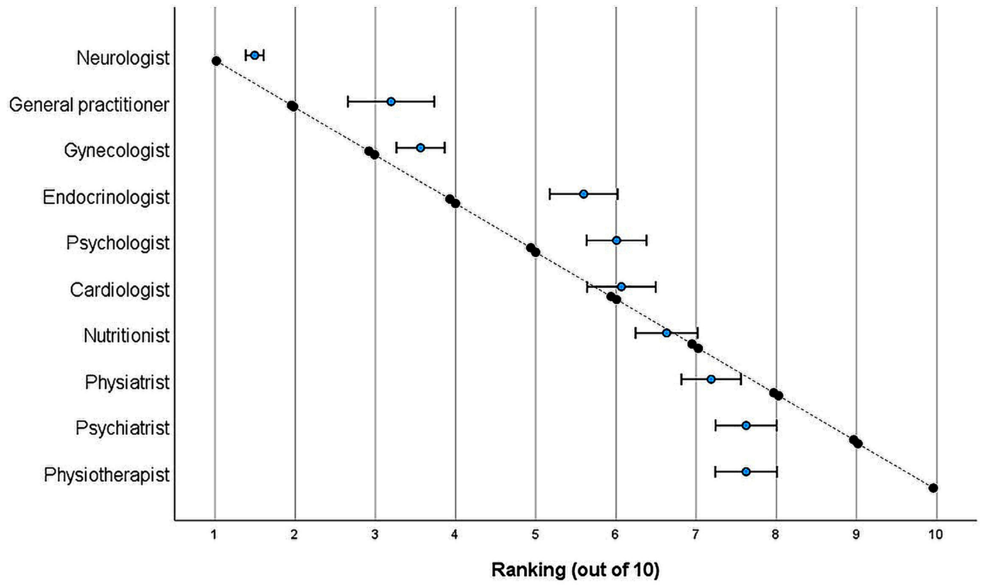

Supplement: Supplementary file 1 [file healthcare-14-02014-s001.zip › Delphi Multidisciplinary Management Migraine_Supplementary material - Figure S1.pdf]
