# Supplementary material for: Multidisciplinary Management of Women Suffering from Migraine: Rationale, Design and Results of a National Delphi Consensus
Source: Healthcare (Basel). 2026 Jul 6;14(13):2014. doi: 10.3390/healthcare14132014 (PMC13361374; doi:10.3390/healthcare14132014)
Supplement: Supplementary file 1 [file healthcare-14-02014-s001.zip › Delphi Multidisciplinary Management Migraine_Supplementary Material - Participants.pdf]

Supplementary Material – Participants to the two-round Delphi process

NEUROLOGISTS
Giorgio Dalla Volta - Brescia
Francesco De Cesaris - Firenze
Roberto De Icco - Pavia
Marina de Tommaso - Bari
Alberto Doretti - Milano
Delfina Ferrandi - Alessandria
Franco Granella - Parma
Simona Guerzoni - Modena
Rosario Iannacchero - Catanzaro
Luigi Francesco Iannone - Firenze
Edoardo Mampreso - Padova
Raffaele Ornello - L'Aquila
Maria Pia Prudenzano - Bari
Marina Romozzi - Roma
Eugenia Rota - Novi Ligure
Elisa Rubino - Torino
Antonio Russo - Napoli
Grazia Sances - Pavia
Paola Sarchielli - Perugia
Gabriele Sebastianelli - Roma
Cristina Tassorelli - Pavia
Irene Toldo - Padova
Gloria Vaghi - Pavia
Fabrizio Vernieri - Roma
Giovanna Viticchi - Ancona
Maria Albanese - Roma
Gennaro Alfieri - Capodrise
Giovanni Battista Allais - Torino
Massimo Autunno - Pellaro
Monica Bandettini di Poggio - Genova
Luca Bartolini - Firenze
Maria Letizia Bartolozzi - Firenze
Laura Borrello - Roma
Simone Braca - Napoli
Antonio Carnevale - Roma
Ilaria Cetta - Milano
Bruno Colombo - Milano
Francesca Cortese - Milano
Fabrizio Di Stani - Roma
Valeria Drago - Siracusa
Valentina Favoni - Bologna
Ludovica Ferraù - Sant'Agata Militello
Cinzia Finocchi - Genova
Francesca Gragnani - Roma
Enrico Grassi - Prato
Emanuele Marcia - Cagliari
Marilena Marcosano - Roma
Davide Mascarella - Bologna
Claudia Anna Mastroeni - Messina
Alessandro Mechelli - Ferrara
Roberta Messina - Milano
Angelo Miele - Napoli
Danilo Antonio Montisano - Milano
Valentina Oppo - Cagliari
Daniele Pala - Cagliari
Francesco Perini - Cologna Veneta VR
Francesca Pistoia - L'Aquila
Angelo Ranieri - Terzigno
Antonio Salerno - Roma
Mattia Sansone - Napoli
Paola Torelli - Parma
Rosario Vecchio - Linguaglossa
Maurizio Zucco - Roma

CARDIOLOGISTS
Natale Daniele Brunetti - Foggia
Matteo Cameli - Siena
Stefano Carugo - Milano
Emilia D'Elia - Bergamo
Federica Ilardi - Napoli
Anna Vittoria Mattioli - Bologna
Roberta Montisci - Cagliari
Saverio Muscoli - Roma
Ermanno Nardi - Napoli
Savina Nodari - Brescia
Stefania Paolillo - Napoli
Giuseppe Patti - Novara
Alberto Polimeni - Catanzaro
Paolo Severino - Roma
Carmen Anna Maria Spaccarotella - Napoli

ENDOCRINOLOGISTS
Gianluca Aimaretti - Novara
Renata Simona Auriemma - Napoli
Giuseppe Bellastella - Napoli
Mara Boschetti - Genova
Salvatore Cannavò - Messina
Massimiliano Caprio - Roma
Guendalina Del Vecchio - Napoli
Luigi di Filippo - Milano
Antonella Di Sarno - Napoli
Carolina Di Somma - Napoli
Katherine Esposito - Napoli
Chiara Graziadio - Roma
Francesco Lombardo - Roma
Giovanna Mantovani - Milano
Paolo Marzullo - Novara
Silvia Migliaccio - Roma
Salvatore Monti - Roma
Giuseppe Pasimeni - Roma
Rosario Pivonello - Napoli
Maurizio Poggi - Roma
Flavia Prodam - Torino
Paola Razzore - Torino
Roberta Scairati - Napoli
Manuela Simoni - Modena

GENERAL PRACTITIONERS
Lora Accettura - Modugno BA
Domenico Aloia - Mola di Bari
Lucia Casatta - Udine
Stefano Celotto - Udine
Giulia Ciancarella - Roma
Corinna Garaffa - Napoli
Arianna Madeo - Erchie BR
Lucia Marasciulo - Bari
Giulio Nati - Roma
Loris Pagano - Roma
Marco Prastaro - Belvedere Marittimo
Andrea Salvetti - Grosseto
Pietro Tasegian - Perugia
Chiara Villani - Taranto

GYNECOLOGISTS
Francesca Albani - Pavia
Federica Battista - Milano
Benedetta Brundu - Piacenza
Giampiero Capobianco - Sassari
Pasquale De Franciscis - Napoli
Davide Dealberti - Alessandria
Silvia Detaddei - Vercelli
Costantino Di Carlo - Napoli
Manuela Farris - Roma
Francesca Ferdeghini - Legnano
Marco Gambacciani - Pisa
Andrea Genazzani - Siena
Giovanni Grandi - Modena
Maurizio Guida - Salerno
Donald Lorenzi - Voghera (PV)
Stefano Luisi - Pisa
Antonio Maiorana - Palermo
Maria Cristina Meriggiola - Bologna
Pietro Molinaro - Roma
Luigi Nappi - Foggia
Francesca Nocera - Catania
Alessandra Ornati - Pavia
Arianna Pacchiarotti - Roma
Anna Maria Paoletti - Cagliari
Felice Petraglia - Firenze
Manuela Piccinino - Pavia
Gianfranco Quintarelli - Prato
Roberta Rossini - Pavia
Valentina Santamaria - Cremona
Tommaso Simoncini - Pisa
Arianna Sommacal - Milano
Giulia Stincardini - Trieste
Massimo Stomati - Brindisi
Lara Tiranini - Pavia
Silvia Tonani - Torino
Marco Torella - Napoli
Mario Vicino - Bari
