# Supplementary material for: Multidisciplinary Management of Women Suffering from Migraine: Rationale, Design and Results of a National Delphi Consensus
Source: Healthcare (Basel). 2026 Jul 6;14(13):2014. doi: 10.3390/healthcare14132014 (PMC13361374; doi:10.3390/healthcare14132014)
Supplement: Supplementary file 1 [file healthcare-14-02014-s001.zip › Delphi Multidisciplinary Management Migraine_Supplementary material - Table S1-S6.pdf]

Supplementary Material - Table S1. Migraine management distribution

		Cardiology		Endocrinology		Gynecology		Neurology		General Practice	
		n	%	n	%	n	%	n	%	n	%
How many migraine cases do you see in a year?	10-50	11	79%	19	73%	23	72%	1	2%	9	69%
	51-100	2	14%	5	19%	5	16%	3	5%	2	15%
	>100	1	7%	2	8%	4	13%	56	93%	2	15%
How many of these patients are women?	25%	0	0%	0	0%	1	3%	0	0%	2	15%
	50%	3	21%	3	12%	0	0%	2	3%	0	0%
	75%	9	64%	15	58%	2	6%	44	73%	9	69%
	>=90%	2	14%	8	31%	29	91%	14	23%	2	15%
How are the women with migraine you see in your clinical practice distributed among the following age groups?	14-18 y		2%		7%		10%		10%		9%
	19-30 y		18%		31%		33%		28%		27%
	31-50 y		38%		38%		40%		37%		37%
	50-60 y		31%		18%		14%		17%		18%
	60+ y		10%		6%		4%		9%		9%
How are the women with migraine you see in your clinical practice distributed among the following categories?	In childbearing age unrelated to menstruation		26%		29%		22%		35%		40%
	In childbearing age with migraine attacks related to menstruation		36%		47%		55%		47%		39%
	In peri/menopausal age		38%		24%		23%		18%		21%
Does your facility have a migraine treatment center?	Yes	12	86%	25	96%	19	59%	59	98%	7	54%
	No	2	14%	1	4%	13	41%	1	2%	6	46%
Are there doctors in your facility who are experts in the diagnosis and treatment of migraines?	Yes	12	86%	25	96%	22	69%	60	100%	6	46%
	No	2	14%	1	4%	10	31%	0	0%	7	54%
What interactions are there between the neurologist and other specialties (endocrinologist, cardiologist, gynecologist)?	None	1	7%	3	12%	2	6%	11	18%	6	46%
	Standardized teamwork	0	0%	0	0%	1	3%	2	3%	0	0%
	Occasional consultation with individual specialists for more severe cases	12	86%	19	73%	25	78%	41	68%	6	46%
	Regular sharing of clinical information for a coordinated approach to diagnosis and treatment	1	7%	4	15%	4	13%	6	10%	1	8%
What is the relationship between specialists and general practitioners regarding this pathology?	None	5	36%	10	38%	9	28%	8	13%	2	15%
	Only unstructured, extemporaneous reports	8	57%	13	50%	18	56%	33	55%	9	69%
	Referral of the patient whose migraine presents defined characteristics	1	7%	3	12%	5	16%	15	25%	2	15%
	Structured sharing of clinical information and coordination of therapies	0	0%	0	0%	0	0%	4	7%	0	0%
Diagnostic and Therapeutic Care Pathway in the clinical center	Yes	1	7%	6	23%	3	9%	30	50%	2	15%
	No	13	93%	20	77%	29	91%	30	50%	11	85%
Diagnostic and Therapeutic Care Pathway in the region	Yes	1	7%	8	31%	11	34%	34	57%	3	23%
	No	13	93%	18	69%	21	66%	26	43%	10	77%

Supplementary Material - Table S2. Consensus Q1 – Topic PUBERTY AND MENSTRUAL

Topic	question	item description	All	All average	cardiology	endocrinology	ginecology	neurology	GP
Puberty and menstrual	1. For a girl of pubertal age who begins to suffer from recurrent attacks of headache with migraine characteristics, it is necessary to request:	No further investigation	inappropriate	inappropriate	inappropriate	inappropriate	inappropriate	inappropriate	uncertain
		Hormone testing	uncertain	uncertain	appropriate	appropriate	uncertain	inappropriate	appropriate
		Brain MRI	uncertain	uncertain	uncertain	uncertain	uncertain	inappropriate	uncertain
		Neurological examination	appropriate	appropriate	appropriate	appropriate	appropriate	appropriate	appropriate
		Headache diary	appropriate	appropriate	appropriate	appropriate	appropriate	appropriate	appropriate
		Gynecological examination	uncertain	uncertain	uncertain	uncertain	appropriate	uncertain	uncertain
		Psychological assessment	uncertain	uncertain	uncertain	uncertain	uncertain	uncertain	uncertain
		General blood tests	uncertain	uncertain	appropriate	appropriate	uncertain	uncertain	appropriate
		Ear, nose, and throat examination	inappropriate	inappropriate	uncertain	uncertain	uncertain	inappropriate	inappropriate
		Eye examination	uncertain	uncertain	uncertain	appropriate	uncertain	inappropriate	uncertain
		Dental examination	uncertain	uncertain	uncertain	uncertain	uncertain	inappropriate	uncertain
	2. For a girl of pubertal age who begins to suffer from recurrent attacks of headache with migraine characteristics, it is necessary to investigate:	Eating habits	appropriate	appropriate	appropriate	appropriate	appropriate	appropriate	appropriate
		Physical activity/sports	appropriate	appropriate	uncertain	appropriate	appropriate	appropriate	appropriate
		Nighttime use of electronic devices (cell phones, video games)	appropriate	appropriate	appropriate	appropriate	appropriate	appropriate	appropriate
		Sleep quality	appropriate	appropriate	appropriate	appropriate	appropriate	appropriate	appropriate
		Use of addictive substances	appropriate	appropriate	appropriate	appropriate	appropriate	appropriate	appropriate
		Family history	appropriate	appropriate	appropriate	appropriate	appropriate	appropriate	appropriate
		Body Mass Index	appropriate	appropriate	appropriate	appropriate	uncertain	appropriate	appropriate
	3. In the case of menstrual-related headaches, it is necessary to:	No further investigation	inappropriate	inappropriate	inappropriate	inappropriate	inappropriate	inappropriate	inappropriate
		Hormone testing	uncertain	uncertain	appropriate	appropriate	appropriate	inappropriate	appropriate
		Brain MRI	uncertain	uncertain	uncertain	uncertain	uncertain	inappropriate	uncertain
		ICHD-3 diagnosis of headache type	appropriate	appropriate	appropriate	appropriate	appropriate	appropriate	appropriate
		Headache diary	appropriate	appropriate	appropriate	appropriate	appropriate	appropriate	appropriate
		Gynecological history/contraception	appropriate	appropriate	appropriate	appropriate	appropriate	appropriate	appropriate
		Pelvic ultrasound	uncertain	uncertain	uncertain	appropriate	appropriate	inappropriate	appropriate
		Gynecological examination	appropriate	appropriate	appropriate	appropriate	appropriate	uncertain	appropriate
		Endocrinological examination	uncertain	uncertain	uncertain	appropriate	uncertain	inappropriate	uncertain
	4. Treatment of menstrual migraine:	is a neurological issue	appropriate	uncertain	appropriate	appropriate	appropriate	appropriate	uncertain
		is a gynecological issue	uncertain	uncertain	uncertain	uncertain	appropriate	uncertain	uncertain
		is a general practitioner issue	uncertain	uncertain	uncertain	uncertain	uncertain	inappropriate	appropriate
		requires collaboration between multiple specialists	appropriate	appropriate	appropriate	appropriate	appropriate	appropriate	uncertain
		requires hormone therapy	uncertain	uncertain	uncertain	uncertain	appropriate	uncertain	uncertain
	5. Hormonal contraceptive therapy is the first approach for women suffering from menstrual migraine.		uncertain	uncertain	uncertain	uncertain	appropriate	inappropriate	uncertain

Besides the overall agreement, which is a weighted average (consequently biased toward opinions of more represented specialties), we computed also a simple (arithmetic) average with equal weights for each specialty (1/5).

Supplementary Material - Table S3. Consensus Q1 – Topic HORMONAL CONTRACEPTION

Topic	question	item description	All	All average	cardiology	endocrinology	ginecology	neurology	GP
**Hormonal Contraception**	6. The presence of migraine should always be assessed during the initial consultation with women who require hormonal contraception.		appropriate	appropriate	appropriate	appropriate	appropriate	appropriate	appropriate
	7. Symptoms attributable to migraine aura should always be assessed during the initial consultation with women who require hormonal contraception.		appropriate	appropriate	appropriate	appropriate	appropriate	appropriate	appropriate
	8. Ischemic risk in women with migraine is a parameter to be assessed in the overall management of the patient:	Always	appropriate	appropriate	appropriate	appropriate	appropriate	appropriate	appropriate
		Only in patients with a family history	uncertain	uncertain	uncertain	uncertain	uncertain	inappropriate	uncertain
		In patients with migraine aura	appropriate	appropriate	appropriate	appropriate	appropriate	appropriate	appropriate
		In patients with risk factors/unhealthy lifestyle	appropriate	appropriate	appropriate	appropriate	appropriate	appropriate	appropriate
	9. A woman suffering from migraine who is undergoing estrogen-progestin therapy needs:	Gynecological or neurological follow-up only	uncertain	uncertain	uncertain	uncertain	uncertain	uncertain	uncertain
		Thrombophilia screening	appropriate	appropriate	uncertain	appropriate	uncertain	uncertain	appropriate
		Monitoring for the possible onset of aura	appropriate	appropriate	appropriate	appropriate	appropriate	appropriate	appropriate
		Headache diary	appropriate	appropriate	appropriate	appropriate	appropriate	appropriate	appropriate
		Cardiological examination	uncertain	uncertain	uncertain	uncertain	uncertain	inappropriate	uncertain
	10. The presence of migraine should always be assessed during the initial assessment of women with gynecological conditions requiring hormone therapy (e.g., endometriosis).		appropriate	appropriate	appropriate	appropriate	appropriate	appropriate	appropriate
	11. The presence of gynecological, metabolic, or hormonal comorbidities should always be assessed during the initial assessment of women with migraine in order to refer the patient to a multidisciplinary approach.		appropriate	appropriate	appropriate	appropriate	appropriate	appropriate	appropriate
	12. Women with migraine and a history of polycystic ovary syndrome should undergo further diagnostic testing of their glucose and lipid metabolism.		appropriate	appropriate	appropriate	appropriate	appropriate	appropriate	appropriate
	13. The presence of cardiovascular comorbidities should always be assessed when first treating women with migraine who require specific therapy.		appropriate	appropriate	appropriate	appropriate	appropriate	appropriate	appropriate

Supplementary Material - Table S4. Consensus Q1 – Topic PREGNANCY AND BREASTFEEDING

Topic	question	item description	All	All average	cardiology	endocrinology	ginecology	neurology	GP
**Pregnancy and Breastfeeding**	15. A woman with migraines who is trying to conceive needs:	Only gynecological or neurological follow-up	uncertain	uncertain	uncertain	inappropriate	uncertain	uncertain	uncertain
		Always plan pregnancy in advance	uncertain	uncertain	uncertain	uncertain	uncertain	uncertain	inappropriate
		Plan pregnancy in advance	appropriate	appropriate	appropriate	appropriate	appropriate	appropriate	appropriate
		Training on managing migraine attacks during pregnancy	appropriate	appropriate	appropriate	appropriate	appropriate	appropriate	appropriate
	16. Migraine during pregnancy is not a problem because it tends to resolve spontaneously.		uncertain	inappropriate	inappropriate	inappropriate	uncertain	uncertain	uncertain
	17. A woman suffering from migraine, who also experiences attacks during pregnancy, needs:	Follow-up only	inappropriate	inappropriate	inappropriate	inappropriate	inappropriate	inappropriate	uncertain
		Neurological assessment	appropriate	appropriate	appropriate	appropriate	appropriate	appropriate	appropriate
		Blood pressure monitoring	appropriate	appropriate	appropriate	appropriate	appropriate	appropriate	appropriate
		Blood pressure monitoring and proteinuria	appropriate	appropriate	appropriate	appropriate	appropriate	appropriate	appropriate
	18. Migraine prophylaxis during pregnancy:	Not necessary	inappropriate	inappropriate	inappropriate	inappropriate	inappropriate	inappropriate	inappropriate
		Always contraindicated	inappropriate	inappropriate	inappropriate	inappropriate	inappropriate	inappropriate	inappropriate
		Uses non-pharmacological therapy (e.g., acupuncture, mindfulness)	appropriate	appropriate	appropriate	appropriate	appropriate	appropriate	appropriate
		Uses the same medications prescribed outside of pregnancy	inappropriate	inappropriate	inappropriate	inappropriate	inappropriate	inappropriate	inappropriate
	19. A woman suffering from migraine during pregnancy needs:	Gynecological follow-up only	uncertain	uncertain	uncertain	inappropriate	uncertain	inappropriate	uncertain
		Neurological follow-up if requested by the gynecologist	appropriate	uncertain	appropriate	uncertain	appropriate	uncertain	appropriate
		Always a neurological follow-up	appropriate	uncertain	uncertain	uncertain	appropriate	appropriate	uncertain
		Headache diary	appropriate	appropriate	appropriate	appropriate	appropriate	appropriate	appropriate
	20. A woman suffering from migraine who wants to undergo ART needs:	Gynecological follow-up only	inappropriate	inappropriate	inappropriate	inappropriate	inappropriate	inappropriate	inappropriate
		Neurological follow-up if requested by the gynecologist	appropriate	appropriate	appropriate	appropriate	appropriate	appropriate	appropriate
		Always a neurological follow-up	appropriate	appropriate	appropriate	appropriate	appropriate	appropriate	appropriate
		Headache diary	inappropriate	inappropriate	inappropriate	inappropriate	inappropriate	inappropriate	uncertain
	21. A woman suffering from migraine who is breastfeeding needs:	only follow-up by the general practitioner	inappropriate	inappropriate	inappropriate	inappropriate	inappropriate	inappropriate	uncertain
		to stop breastfeeding in case of frequency	uncertain	uncertain	uncertain	inappropriate	uncertain	uncertain	uncertain
		a multidisciplinary approach (neurologist, pediatrician, gynecologist, endocrinologist)	appropriate	appropriate	appropriate	appropriate	appropriate	appropriate	appropriate
		advance training on seizure management	appropriate	appropriate	appropriate	appropriate	appropriate	appropriate	appropriate

Supplementary Material - Table S5. Consensus Q1 – Topic MENOPAUSE

**Topic**	**question**	**item description**	All	All average	cardiology	endocrinology	ginecology	neurology	GP
**Menopause**	22. Migraines worsen during the transition from pre- to post-menopause.		uncertain	uncertain	uncertain	uncertain	uncertain	uncertain	uncertain
	23. Migraine improves in the post-menopausal phase.		appropriate	uncertain	appropriate	appropriate	appropriate	uncertain	appropriate
	24. Women suffering from migraine require greater cardiovascular attention during the transition from pre- to post-menopause.		appropriate	appropriate	appropriate	appropriate	appropriate	appropriate	appropriate
	25. Hormone replacement therapy in menopausal women with migraine:	can be prescribed early in asymptomatic patients.	uncertain	uncertain	inappropriate	uncertain	uncertain	inappropriate	uncertain
		can be prescribed early only in symptomatic patients.	uncertain	uncertain	uncertain	uncertain	uncertain	uncertain	uncertain
		should never be prescribed.	inappropriate	inappropriate	inappropriate	inappropriate	inappropriate	inappropriate	inappropriate
		can be prescribed but with monitoring via a headache diary.	appropriate	appropriate	appropriate	appropriate	appropriate	appropriate	appropriate
		must be personalized.	appropriate	appropriate	appropriate	appropriate	appropriate	appropriate	appropriate
	28. The first level of intervention for the prevention of cardiovascular and cerebrovascular disease in postmenopausal women with migraine includes:	Diet and exercise	appropriate	appropriate	appropriate	appropriate	appropriate	appropriate	appropriate
		Prescription of specialist tests	appropriate	uncertain	appropriate	uncertain	appropriate	uncertain	appropriate
		Hormone replacement therapy in women with post-menopausal symptoms	uncertain	uncertain	uncertain	uncertain	appropriate	uncertain	uncertain
		Reduction in the frequency of migraine attacks	appropriate	appropriate	appropriate	appropriate	appropriate	appropriate	appropriate
	29. The age at menopause influences the natural history of migraine.		appropriate	appropriate	appropriate	appropriate	appropriate	uncertain	appropriate
	30. The type of menopause (physiological, surgical, pharmacological) influences the natural history of migraine.		appropriate	appropriate	appropriate	appropriate	appropriate	appropriate	appropriate

Supplementary Material - Table S6. Consensus Q1 – Topic DIAGNOSIS AND STIGMATIZATION

**Topic**	**question**	**item description**	All	All average	cardiology	endocrinology	ginecology	neurology	GP
**Diagnosis and Stigmatization**	31. The priority target for managing a woman with migraine should include:	Prevention of cardiovascular disease	appropriate	appropriate	appropriate	appropriate	appropriate	appropriate	appropriate
		Reduction of disability related to attacks	appropriate	appropriate	appropriate	appropriate	appropriate	appropriate	appropriate
		Reduction of inter-critical disability	appropriate	appropriate	appropriate	appropriate	appropriate	appropriate	appropriate
		Reduction in the monthly number of attacks	appropriate	appropriate	appropriate	appropriate	appropriate	appropriate	appropriate
		Prevention of complications from analgesic use	appropriate	appropriate	appropriate	appropriate	appropriate	appropriate	appropriate
	32. Which healthcare professional should a woman suffering from migraine consult?	General practitioner	appropriate	appropriate	appropriate	uncertain	appropriate	appropriate	appropriate
		Gynecologist	appropriate	appropriate	appropriate	appropriate	appropriate	uncertain	appropriate
		Endocrinologist	uncertain	uncertain	uncertain	appropriate	uncertain	inappropriate	uncertain
		Cardiologist	uncertain	uncertain	appropriate	appropriate	appropriate	uncertain	uncertain
		Neurologist	appropriate	appropriate	appropriate	appropriate	appropriate	appropriate	appropriate
		Psychiatrist	uncertain	uncertain	uncertain	uncertain	uncertain	uncertain	uncertain
		Psychologist	uncertain	uncertain	uncertain	uncertain	uncertain	uncertain	uncertain
		Physiatrist	uncertain	uncertain	uncertain	uncertain	uncertain	inappropriate	uncertain
		Physical therapist	uncertain	uncertain	uncertain	uncertain	uncertain	uncertain	uncertain
		Nutritionist	uncertain	uncertain	uncertain	uncertain	uncertain	uncertain	uncertain
	33. To reduce the stigma affecting women with migraine, it is necessary to:	Nothing, migraine is no longer a stigma	inappropriate	inappropriate	inappropriate	inappropriate	inappropriate	inappropriate	inappropriate
		Education in schools	appropriate	appropriate	appropriate	appropriate	appropriate	appropriate	appropriate
		Media campaigns	appropriate	appropriate	appropriate	appropriate	appropriate	appropriate	appropriate
		Involvement of the world of work	appropriate	appropriate	appropriate	appropriate	appropriate	appropriate	appropriate
		Training of the medical profession	appropriate	appropriate	appropriate	appropriate	appropriate	appropriate	appropriate
		Involvement of institutions	appropriate	appropriate	appropriate	appropriate	appropriate	appropriate	appropriate
		Involvement of social media	appropriate	appropriate	appropriate	appropriate	appropriate	appropriate	appropriate
	34. The primary objective of an information campaign on migraine should be:	Recognition of migraine as a neurological disorder	appropriate	appropriate	appropriate	appropriate	appropriate	appropriate	appropriate
		Control/training in relation to self-medication	appropriate	appropriate	appropriate	appropriate	appropriate	appropriate	appropriate
		Indicating the correct course of treatment	appropriate	appropriate	appropriate	appropriate	appropriate	appropriate	appropriate
		Recognition of migraine as a disabling condition, especially for women	appropriate	appropriate	appropriate	appropriate	appropriate	appropriate	appropriate
		Dispelling false myths (e.g., cervical headache)	appropriate	appropriate	appropriate	appropriate	appropriate	appropriate	appropriate
		Prevention through healthy lifestyles	appropriate	appropriate	appropriate	appropriate	appropriate	appropriate	appropriate
		Promotion of specialized migraine treatment centers nationwide	appropriate	appropriate	appropriate	appropriate	appropriate	appropriate	appropriate
