# Supplementary material for: Multidisciplinary Management of Women Suffering from Migraine: Rationale, Design and Results of a National Delphi Consensus
Source: Healthcare (Basel). 2026 Jul 6;14(13):2014. doi: 10.3390/healthcare14132014 (PMC13361374; doi:10.3390/healthcare14132014)
Supplement: Supplementary file 1 [file healthcare-14-02014-s001.zip › Delphi Multidisciplinary Management Migraine_Supplementary material - Table S7.pdf]

Supplementary Material - Table S7. All the statements with corresponding agreement levels across both rounds of the Delphi process.

			all specialties																				
question		item	ratings	mean	median	SD	range	min	max	p25	p30	p50	p70	p75	IQR	I_P30_P70_R	IPRCP	AI	IPRAS	disagreement	appropriateness	appropriateness/agreement	evaluation
1. For a girl of pubertal age who begins to suffer from recurrent attacks of headache with migraine characteristics, it is necessary to request:	No further investigation	d01_r01	145	2,2	1,0	2,2	8,0	1,0	9,0	1,0	1,0	1,0	2,0	2,5	1,5	1,0	1,5	3,5	7,6	0	-1	-1	inappropriate
	Hormone testing	d01_r02	145	4,7	5,0	2,7	8,0	1,0	9,0	2,0	2,0	5,0	7,0	7,0	5,0	5,0	4,5	0,5	3,1	1	0	10	uncertain
	Brain MRI	d01_r03	145	4,4	4,0	2,6	8,0	1,0	9,0	2,0	3,0	4,0	6,0	7,0	5,0	3,0	4,5	0,5	3,1	0	0	0	uncertain
	Neurological examination	d01_r04	145	7,9	9,0	1,7	8,0	1,0	9,0	7,0	8,0	9,0	9,0	9,0	2,0	1,0	8,5	3,5	7,6	0	1	1	appropriate
	Headache diary	d01_r05	145	8,4	9,0	1,2	8,0	1,0	9,0	8,0	8,8	9,0	9,0	9,0	1,0	0,2	8,9	3,9	8,2	0	1	1	appropriate
	Gynecological examination	d01_r06	145	5,1	5,0	2,4	8,0	1,0	9,0	3,0	4,0	5,0	7,0	7,0	4,0	3,0	5,5	0,5	3,1	0	0	0	uncertain
	Psychological assessment	d01_r07	145	4,7	5,0	2,1	8,0	1,0	9,0	3,0	3,0	5,0	6,0	6,0	3,0	3,0	4,5	0,5	3,1	0	0	0	uncertain
	General blood tests	d01_r08	145	6,0	6,0	2,4	8,0	1,0	9,0	5,0	5,0	6,0	7,0	8,0	3,0	2,0	6,0	1,0	3,9	0	0	0	uncertain
	Ear, nose, and throat examination	d01_r09	145	3,3	2,0	2,4	8,0	1,0	9,0	1,0	1,0	2,0	5,0	5,0	4,0	4,0	3,0	2,0	5,4	0	-1	-1	inappropriate
	Eye examination	d01_r10	145	4,7	5,0	2,7	8,0	1,0	9,0	2,0	3,0	5,0	7,0	7,0	5,0	4,0	5,0	0,0	2,4	1	0	10	uncertain
	Dental examination	d01_r11	145	3,8	3,0	2,6	8,0	1,0	9,0	1,0	1,0	3,0	6,0	6,0	5,0	5,0	3,5	1,5	4,6	1	-1	9	uncertain
2. For a girl of pubertal age who begins to suffer from recurrent attacks of headache with migraine characteristics, it is necessary to investigate:	Eating habits	d02_r01	145	7,1	8,0	1,9	8,0	1,0	9,0	6,0	7,0	8,0	8,0	9,0	3,0	1,0	7,5	2,5	6,1	0	1	1	appropriate
	Physical activity/sports	d02_r02	145	7,1	7,0	1,8	7,0	2,0	9,0	6,0	6,0	7,0	8,0	8,5	2,5	2,0	7,0	2,0	5,4	0	1	1	appropriate
	Nighttime use of electronic devices (cell phones, video games)	d02_r03	145	7,2	8,0	2,2	8,0	1,0	9,0	6,0	6,0	8,0	9,0	9,0	3,0	3,0	7,5	2,5	6,1	0	1	1	appropriate
	Sleep quality	d02_r04	145	8,1	9,0	1,3	7,0	2,0	9,0	8,0	8,0	9,0	9,0	9,0	1,0	1,0	8,5	3,5	7,6	0	1	1	appropriate
	Use of addictive substances	d02_r05	145	7,7	8,0	1,7	8,0	1,0	9,0	7,0	7,0	8,0	9,0	9,0	2,0	2,0	8,0	3,0	6,9	0	1	1	appropriate
	Family history	d02_r06	145	8,1	9,0	1,4	8,0	1,0	9,0	8,0	8,0	9,0	9,0	9,0	1,0	1,0	8,5	3,5	7,6	0	1	1	appropriate
	Body Mass Index	d02_r07	145	7,1	7,0	1,8	8,0	1,0	9,0	6,0	6,0	7,0	8,0	9,0	3,0	2,0	7,0	2,0	5,4	0	1	1	appropriate
3. In the case of menstrual-related headaches, it is necessary to:	No further investigation	d03_r01	145	2,2	1,0	2,1	8,0	1,0	9,0	1,0	1,0	1,0	2,0	3,0	2,0	1,0	1,5	3,5	7,6	0	-1	-1	inappropriate
	Hormone testing	d03_r02	145	5,5	6,0	2,7	8,0	1,0	9,0	3,0	4,0	6,0	7,0	8,0	5,0	3,0	5,5	0,5	3,1	0	0	0	uncertain
	Brain MRI	d03_r03	145	4,0	4,0	2,5	8,0	1,0	9,0	1,5	2,0	4,0	5,2	6,0	4,5	3,2	3,6	1,4	4,5	0	0	0	uncertain
	ICHD-3 diagnosis of headache type	d03_r04	145	8,0	9,0	1,6	8,0	1,0	9,0	7,0	8,0	9,0	9,0	9,0	2,0	1,0	8,5	3,5	7,6	0	1	1	appropriate
	Headache diary	d03_r05	145	8,5	9,0	1,0	5,0	4,0	9,0	8,0	8,0	9,0	9,0	9,0	1,0	1,0	8,5	3,5	7,6	0	1	1	appropriate
	Gynecological history/contraception	d03_r06	145	8,1	9,0	1,4	8,0	1,0	9,0	7,5	8,0	9,0	9,0	9,0	1,5	1,0	8,5	3,5	7,6	0	1	1	appropriate
	Pelvic ultrasound	d03_r07	145	4,8	5,0	2,7	8,0	1,0	9,0	2,0	2,0	5,0	7,0	7,0	5,0	5,0	4,5	0,5	3,1	1	0	10	uncertain
	Gynecological examination	d03_r08	145	6,5	7,0	2,4	8,0	1,0	9,0	5,0	5,0	7,0	8,0	9,0	4,0	3,0	6,5	1,5	4,6	0	1	1	appropriate
	Endocrinological examination	d03_r09	145	5,0	5,0	2,7	8,0	1,0	9,0	2,0	3,0	5,0	7,0	7,0	5,0	4,0	5,0	0,0	2,4	1	0	10	uncertain
4. Treatment of menstrual migraine:	is a neurological issue	d04_r01	145	6,7	7,0	2,3	8,0	1,0	9,0	6,0	6,0	7,0	8,0	8,0	2,0	2,0	7,0	2,0	5,4	0	1	1	appropriate
	is a gynecological issue	d04_r02	145	5,4	6,0	2,6	8,0	1,0	9,0	3,0	4,0	6,0	7,0	8,0	5,0	3,0	5,5	0,5	3,1	0	0	0	uncertain
	is a general practitioner issue	d04_r03	145	3,91	4,0	2,3	8,0	1,0	9,0	2,0	2,0	4,0	5,0	6,0	4,0	3,0	3,5	1,5	4,6	0	0	0	uncertain
	requires collaboration between multiple specialists	d04_r04	145	7,57	9,0	2,0	8,0	1,0	9,0	6,5	7,0	9,0	9,0	9,0	2,5	2,0	8,0	3,0	6,9	0	1	1	appropriate
	requires hormone therapy	d04_r05	145	5,06	5,0	2,2	8,0	1,0	9,0	4,0	4,0	5,0	6,2	7,0	3,0	2,2	5,1	0,1	2,5	0	0	0	uncertain
5. Hormonal contraceptive therapy is the first approach for women suffering from menstrual migraine.		d05_r01	145	4,35	5,0	2,5	8,0	1,0	9,0	2,0	2,0	5,0	6,0	6,0	4,0	4,0	4,0	1,0	3,9	1	0	10	uncertain
6. The presence of migraine should always be assessed during the initial consultation with women who require hormonal contraception.		d06_r01	141	7,94	9,0	1,5	7,0	2,0	9,0	7,0	7,0	9,0	9,0	9,0	2,0	2,0	8,0	3,0	6,9	0	1	1	appropriate
7. Symptoms attributable to migraine aura should always be assessed during the initial consultation with women who require hormonal contraception.		d07_r01	141	8,33	9,0	1,2	6,0	3,0	9,0	8,0	8,0	9,0	9,0	9,0	1,0	1,0	8,5	3,5	7,6	0	1	1	appropriate
8. Ischemic risk in women with migraine is a parameter to be assessed in the overall management of the patient:	Always	d08_r01	141	7,31	8,0	2,0	8,0	1,0	9,0	6,0	6,0	8,0	9,0	9,0	3,0	3,0	7,5	2,5	6,1	0	1	1	appropriate
	Only in patients with a family history	d08_r02	141	3,99	4,0	2,7	8,0	1,0	9,0	1,0	1,0	4,0	6,0	6,0	5,0	5,0	3,5	1,5	4,6	1	0	10	uncertain
	In patients with migraine aura	d08_r03	141	8,13	9,0	1,7	8,0	1,0	9,0	8,0	8,0	9,0	9,0	9,0	1,0	1,0	8,5	3,5	7,6	0	1	1	appropriate
	In patients with risk factors/unhealthy lifestyle	d08_r04	141	7,62	9,0	2,1	8,0	1,0	9,0	7,0	8,0	9,0	9,0	9,0	2,0	1,0	8,5	3,5	7,6	0	1	1	appropriate
9. A woman suffering from migraine who is undergoing estrogen-progestin therapy needs:	Gynecological or neurological follow-up only	d09_r01	141	5,35	5,0	2,7	8,0	1,0	9,0	3,0	3,6	5,0	8,0	8,0	5,0	4,4	5,8	0,8	3,6	1	0	10	uncertain
	Thrombophilia screening	d09_r02	141	6,37	7,0	2,6	8,0	1,0	9,0	5,0	5,0	7,0	8,4	9,0	4,0	3,4	6,7	1,7	4,9	0	1	1	appropriate
	Monitoring for the possible onset of aura	d09_r03	141	7,91	9,0	1,8	8,0	1,0	9,0	7,0	8,0	9,0	9,0	9,0	2,0	1,0	8,5	3,5	7,6	0	1	1	appropriate
	Headache diary	d09_r04	141	8,28	9,0	1,4	8,0	1,0	9,0	8,0	8,0	9,0	9,0	9,0	1,0	1,0	8,5	3,5	7,6	0	1	1	appropriate
	Cardiological examination	d09_r05	141	4,29	4,0	2,4	8,0	1,0	9,0	2,0	2,6	4,0	6,0	6,0	4,0	3,4	4,3	0,7	3,4	0	0	0	uncertain
10. The presence of migraine should always be assessed during the initial assessment of women with gynecological conditions requiring hormone therapy (e.g., endometriosis).		d10_r01	141	7,94	9,0	1,4	6,0	3,0	9,0	7,0	7,0	9,0	9,0	9,0	2,0	2,0	8,0	3,0	6,9	0	1	1	appropriate
11. The presence of gynecological, metabolic, or hormonal comorbidities should always be assessed during the initial assessment of women with migraine in order to refer the patient to a multidisciplinary approach.		d11_r01	141	8,27	9,0	1,1	6,0	3,0	9,0	8,0	8,0	9,0	9,0	9,0	1,0	1,0	8,5	3,5	7,6	0	1	1	appropriate
12. Women with migraine and a history of polycystic ovary syndrome should undergo further diagnostic testing of their glucose and lipid metabolism.		d12_r01	141	7,86	8,0	1,4	7,0	2,0	9,0	7,0	7,0	8,0	9,0	9,0	2,0	2,0	8,0	3,0	6,9	0	1	1	appropriate
13. The presence of cardiovascular comorbidities should always be assessed when first treating women with migraine who require specific therapy.		d13_r01	141	7,77	8,0	1,6	8,0	1,0	9,0	7,0	8,0	8,0	9,0	9,0	2,0	1,0	8,5	3,5	7,6	0	1	1	appropriate
15. A woman with migraines who is trying to conceive needs:	Only gynecological or neurological follow-up	d15_r01	139	5,04	5,0	2,4	8,0	1,0	9,0	3,0	3,0	5,0	7,0	7,0	4,0	4,0	5,0	0,0	2,4	1	0	10	uncertain
	Always plan pregnancy in advance	d15_r02	139	5,1	5,0	2,5	8,0	1,0	9,0	3,0	3,0	5,0	7,0	7,0	4,0	4,0	5,0	0,0	2,4	1	0	10	uncertain
	Plan pregnancy in advance	d15_r03	139	7,53	8,0	2,1	8,0	1,0	9,0	7,0	7,0	8,0	9,0	9,0	2,0	2,0	8,0	3,0	6,9	0	1	1	appropriate
	Training on managing seizures during pregnancy	d15_r04	139	8,29	9,0	1,2	8,0	1,0	9,0	8,0	8,0	9,0	9,0	9,0	1,0	1,0	8,5	3,5	7,6	0	1	1	appropriate
16. Migraine during pregnancy is not a problem because it tends to resolve spontaneously.		d16_r01	139	4,06	4,0	2,1	7,0	1,0	8,0	2,0	3,0	4,0	5,0	6,0	4,0	2,0	4,0	1,0	3,9	0	0	0	uncertain
17. A woman suffering from migraine, who also experiences attacks during pregnancy, needs:	Follow-up only	d17_r01	139	3,24	3,0	2,2	8,0	1,0	9,0	1,0	2,0	3,0	4,0	5,0	4,0	2,0	3,0	2,0	5,4	0	-1	-1	inappropriate
	Neurological assessment	d17_r02	139	7,45	8,0	2,0	8,0	1,0	9,0	7,0	7,0	8,0	9,0	9,0	2,0	2,0	8,0	3,0	6,9	0	1	1	appropriate
	Blood pressure monitoring	d17_r03	139	8,33	9,0	1,1	7,0	2,0	9,0	8,0	8,0	9,0	9,0	9,0	1,0	1,0	8,5	3,5	7,6	0	1	1	appropriate
	Blood pressure monitoring and proteinuria	d17_r04	139	8,02	9,0	1,5	7,0	2,0	9,0	8,0	8,0	9,0	9,0	9,0	1,0	1,0	8,5	3,5	7,6	0	1	1	appropriate
18. Migraine prophylaxis during pregnancy:	Not necessary	d18_r01	139	2,17	1,0	2,0	8,0	1,0	9,0	1,0	1,0	1,0	2,0	2,0	1,0	1,0	1,5	3,5	7,6	0	-1	-1	inappropriate
	Always contraindicated	d18_r02	139	2,45	1,0	2,1	8,0	1,0	9,0	1,0	1,0	1,0	3,0	3,0	2,0	2,0	2,0	3,0	6,9	0	-1	-1	inappropriate
	Uses non-pharmacological therapy (e.g., acupuncture, mindfulness)	d18_r03	139	6,88	7,0	2,1	8,0	1,0	9,0	6,0	6,0	7,0	8,0	9,0	3,0	2,0	7,0	2,0	5,4	0	1	1	appropriate
	Uses the same medications prescribed outside of pregnancy	d18_r04	139	2,53	2,0	1,8	6,0	1,0	7,0	1,0	1,0	2,0	3,0	4,0	3,0	2,0	2,0	3,0	6,9	0	-1	-1	inappropriate
19. A woman suffering from migraine during pregnancy needs:	Gynecological follow-up only	d19_r01	139	3,92	4,0	2,5	8,0	1,0	9,0	1,0	2,0	4,0	5,0	6,0	5,0	3,0	3,5	1,5	4,6	0	0	0	uncertain
	Neurological follow-up if requested by the gynecologist	d19_r02	139	6,01	7,0	2,8	8,0	1,0	9,0	4,0	5,0	7,0	8,0	9,0	5,0	3,0	6,5	1,5	4,6	0	1	1	appropriate
	Always a neurological follow-up	d19_r03	139	6,38	7,0	2,4	8,0	1,0	9,0	5,0	5,0	7,0	8,0	9,0	4,0	3,0	6,5	1,5	4,6	0	1	1	appropriate
	Headache diary	d19_r04	139	8,14	9,0	1,5	8,0	1,0	9,0	8,0	8,0	9,0	9,0	9,0	1,0	1,0	8,5	3,5	7,6	0	1	1	appropriate
20. A woman suffering from migraine who wants to undergo ART needs:	Gynecological follow-up only	d20_r01	139	3,14	2,0	2,3	8,0	1,0	9,0	1,0	1,0	2,0	5,0	5,0	4,0	4,0	3,0	2,0	5,4	0	-1	-1	inappropriate
	Neurological follow-up if requested by the gynecologist	d20_r02	139	8,12	9,0	1,4	6,0	3,0	9,0	8,0	8,0	9,0	9,0	9,0	1,0	1,0	8,5	3,5	7,6	0	1	1	appropriate
	Always a neurological follow-up	d20_r03	139	7,4	9,0	2,5	8,0	1,0	9,0	7,0	7,0	9,0	9,0	9,0	2,0	2,0	8,0	3,0	6,9	0	1	1	appropriate
	Headache diary	d20_r04	139	3,06	2,0	2,3	8,0	1,0	9,0	1,0	1,0	2,0	5,0	5,0	4,0	4,0	3,0	2,0	5,4	0	-1	-1	inappropriate
21. A woman suffering from migraine who is breastfeeding needs:	only follow-up by the general practitioner	d21_r01	139	2,96	2,0	2,2	8,0	1,0	9,0	1,0	1,0	2,0	4,0	4,0	3,0	3,0	2,5	2,5	6,1	0	-1	-1	inappropriate
	to stop breastfeeding in case of high frequency of attacks to start a specific therapy	d21_r02	139	4,52	5,0	2,6	8,0	1,0	9,0	2,0	2,0	5,0	6,0	7,0	5,0	4,0	4,0	1,0	3,9	1	0	10	uncertain
	a multidisciplinary approach (neurologist, pediatrician, gynecologist, endocrinologist)	d21_r03	139	7,58	9,0	2,1	8,0	1,0	9,0	7,0	7,0	9,0	9,0	9,0	2,0	2,0	8,0	3,0	6,9	0	1	1	appropriate
	advance training on seizure management	d21_r04	139	8,26	9,0	1,1	4,0	5,0	9,0	8,0	8,0	9,0	9,0	9,0	1,0	1,0	8,5	3,5	7,6	0	1	1	appropriate
22. Migraines worsen during the transition from pre- to post-menopause.		d22_r01	134	5,8	6,0	2,0	8,0	1,0	9,0	5,0	5,0	6,0	7,0	7,0	2,0	2,0	6,0	1,0	3,9	0	0	0	uncertain
23. Migraine improves in the post-menopausal phase.		d23_r01	134	6,55	7,0	1,5	8,0	1,0	9,0	5,0	6,0	7,0	7,0	8,0	3,0	1,0	6,5	1,5	4,6	0	1	1	appropriate
24. Women suffering from migraine require greater cardiovascular attention during the transition from pre- to post-menopause.		d24_r01	134	7,25	7,5	1,7	8,0	1,0	9,0	7,0	7,0	7,5	8,0	9,0	2,0	1,0	7,5	2,5	6,1	0	1	1	appropriate
25. Hormone replacement therapy in menopausal women with migraine:	can be prescribed early in asymptomatic patients.	d25_r01	134	4,17	4,0	2,3	8,0	1,0	9,0	2,0	2,0	4,0	5,0	6,0	4,0	3,0	3,5	1,5	4,6	0	0	0	uncertain
	can be prescribed early only in symptomatic patients.	d25_r02	134	4,55	5,0	2,2	8,0	1,0	9,0	3,0	3,0	5,0	6,0	6,0	3,0	3,0	4,5	0,5	3,1	0	0	0	uncertain
	should never be prescribed.	d25_r03	134	2,13	1,0	1,7	8,0	1,0	9,0	1,0	1,0	1,0	2,0	3,0	2,0	1,0	1,5	3,5	7,6	0	-1	-1	inappropriate
	can be prescribed but with monitoring via a headache diary.	d25_r04	134	7,21	8,0	2,1	8,0	1,0	9,0	6,0	7,0	8,0	9,0	9,0	3,0	2,0	8,0	3,0	6,9	0	1	1	appropriate
	must be personalized.	d25_r05	134	8,4	9,0	0,9	4,0	5,0	9,0	8,0	8,0	9,0	9,0	9,0	1,0	1,0	8,5	3,5	7,6	0	1	1	appropriate
28. The first level of intervention for the prevention of cardiovascular and cerebrovascular disease in postmenopausal women with migraine includes:	Diet and exercise	d28_r01	134	8,28	9,0	1,1	5,0	4,0	9,0	8,0	8,0	9,0	9,0	9,0	1,0	1,0	8,5	3,5	7,6	0	1	1	appropriate
	Prescription of specialist tests	d28_r02	134	6,09	7,0	2,2	8,0	1,0	9,0	5,0	5,0	7,0	8,0	8,0	3,0	3,0	6,5	1,5	4,6	0	1	1	appropriate
	Hormone replacement therapy in women with post-menopausal symptoms	d28_r03	134	5,85	6,0	2,0	8,0	1,0	9,0	5,0	5,0	6,0	7,0	7,0	2,0	2,0	6,0	1,0	3,9	0	0	0	uncertain
	Reduction in the frequency of migraine attacks	d28_r04	134	6,9	7,0	2,0	8,0	1,0	9,0	5,8	6,0	7,0	8,0	9,0	3,3	2,0	7,0	2,0	5,4	0	1	1	appropriate
29. The age at menopause influences the natural history of migraine.		d29_r01	134	6,48	7,0	1,8	8,0	1,0	9,0	5,0	6,0	7,0	8,0	8,0	3,0	2,0	7,0	2,0	5,4	0	1	1	appropriate
30. The type of menopause (physiological, surgical, pharmacological) influences the natural history of migraine.		d30_r01	134	6,84	7,0	2,0	8,0	1,0	9,0	6,0	6,0	7,0	8,0	8,3	2,3	2,0	7,0	2,0	5,4	0	1	1	appropriate
31. The priority target for managing a woman with migraine should include:	Prevention of cardiovascular disease	d31_r01	134	7,66	8,0	1,8	8,0	1,0	9,0	7,0	7,0	8,0	9,0	9,0	2,0	2,0	8,0	3,0	6,9	0	1	1	appropriate
	Reduction of disability related to attacks	d31_r02	134	8,43	9,0	0,9	5,0	4,0	9,0	8,0	8,0	9,0	9,0	9,0	1,0	1,0	8,5	3,5	7,6	0	1	1	appropriate
	Reduction of inter-critical disability	d31_r03	134	8,05	9,0	1,3	6,0	3,0	9,0	7,8	8,0	9,0	9,0	9,0	1,3	1,0	8,5	3,5	7,6	0	1	1	appropriate
	Reduction in the monthly number of attacks	d31_r04	134	8,28	9,0	1,1	5,0	4,0	9,0	8,0	8,0	9,0	9,0	9,0	1,0	1,0	8,5	3,5	7,6	0	1	1	appropriate
	Prevention of complications from analgesic use	d31_r05	134	7,96	8,0	1,3	6,0	3,0	9,0	7,0	7,5	8,0	9,0	9,0	2,0	1,5	8,3	3,3	7,2	0	1	1	appropriate
32. Which healthcare professional should a woman suffering from migraine consult?	General practitioner	d32_r01	134	6,66	7,0	2,3	8,0	1,0	9,0	5,0	6,0	7,0	9,0	9,0	4,0	3,0	7,5	2,5	6,1	0	1	1	appropriate
	Gynecologist	d32_r02	134	6,66	7,0	2,0	8,0	1,0	9,0	5,0	6,0	7,0	8,0	8,0	3,0	2,0	7,0	2,0	5,4	0	1	1	appropriate
	Endocrinologist	d32_r03	134	5,08	5,0	2,4	8,0	1,0	9,0	3,0	3,0	5,0	7,0	7,0	4,0	4,0	5,0	0,0	2,4	1	0	10	uncertain
	Cardiologist	d32_r04	134	5,65	6,0	2,4	8,0	1,0	9,0	4,0	5,0	6,0	7,0	8,0	4,0	2,0	6,0	1,0	3,9	0	0	0	uncertain
	Neurologist	d32_r05	134	8,56	9,0	0,9	5,0	4,0	9,0	8,0	9,0	9,0	9,0	9,0	1,0	0,0	9,0	4,0	8,4	0	1	1	appropriate
	Psychiatrist	d32_r06	134	4,33	5,0	2,1	8,0	1,0	9,0	2,0	3,0	5,0	6,0	6,0	4,0	3,0	4,5	0,5	3,1	0	0	0	uncertain
	Psychologist	d32_r07	134	5,31	6,0	2,1	8,0	1,0	9,0	4,0	5,0	6,0	7,0	7,0	3,0	2,0	6,0	1,0	3,9	0	0	0	uncertain
	Physiatrist	d32_r08	134	4,34	5,0	2,2	7,0	1,0	8,0	2,0	3,0	5,0	6,0	6,0	4,0	3,0	4,5	0,5	3,1	0	0	0	uncertain
	Physical therapist	d32_r09	134	4,31	5,0	2,3	8,0	1,0	9,0	2,0	3,0	5,0	6,0	6,0	4,0	3,0	4,5	0,5	3,1	0	0	0	uncertain
	Nutritionist	d32_r10	134	5,44	6,0	2,2	8,0	1,0	9,0	4,0	4,0	6,0	7,0	7,0	3,0	3,0	5,5	0,5	3,1	0	0	0	uncertain
33. To reduce the stigma affecting women with migraine, it is necessary to:	Nothing, migraine is no longer a stigma	d33_r01	134	2,08	1,0	1,8	8,0	1,0	9,0	1,0	1,0	1,0	2,0	3,0	2,0	1,0	1,5	3,5	7,6	0	-1	-1	inappropriate
	Education in schools	d33_r02	134	7,65	8,0	1,8	8,0	1,0	9,0	7,0	7,0	8,0	9,0	9,0	2,0	2,0	8,0	3,0	6,9	0	1	1	appropriate
	Media campaigns	d33_r03	134	7,6	8,0	1,5	7,0	2,0	9,0	7,0	7,0	8,0	9,0	9,0	2,0	2,0	8,0	3,0	6,9	0	1	1	appropriate
	Involvement of the world of work	d33_r04	134	7,86	8,0	1,4	7,0	2,0	9,0	7,0	7,5	8,0	9,0	9,0	2,0	1,5	8,3	3,3	7,2	0	1	1	appropriate
	Training of the medical profession	d33_r05	134	8,34	9,0	1,2	7,0	2,0	9,0	8,0	8,0	9,0	9,0	9,0	1,0	1,0	8,5	3,5	7,6	0	1	1	appropriate
	Involvement of institutions	d33_r06	134	7,79	8,0	1,6	8,0	1,0	9,0	7,0	7,0	8,0	9,0	9,0	2,0	2,0	8,0	3,0	6,9	0	1	1	appropriate
	Involvement of social media	d33_r07	134	7,57	8,0	1,5	7,0	2,0	9,0	7,0	7,0	8,0	9,0	9,0	2,0	2,0	8,0	3,0	6,9	0	1	1	appropriate
34. The primary objective of an information campaign on migraine should be:	Recognition of migraine as a neurological disorder	d34_r01	134	8,22	9,0	1,3	5,0	4,0	9,0	8,0	8,0	9,0	9,0	9,0	1,0	1,0	8,5	3,5	7,6	0	1	1	appropriate
	Control/training in relation to self-medication	d34_r02	134	7,85	8,0	1,6	8,0	1,0	9,0	7,0	7,5	8,0	9,0	9,0	2,0	1,5	8,3	3,3	7,2	0	1	1	appropriate
	Indicating the correct course of treatment	d34_r03	134	8,47	9,0	0,9	5,0	4,0	9,0	8,0	8,0	9,0	9,0	9,0	1,0	1,0	8,5	3,5	7,6	0	1	1	appropriate
	Recognition of migraine as a disabling condition, especially for women	d34_r04	134	8,23	9,0	1,2	6,0	3,0	9,0	8,0	8,0	9,0	9,0	9,0	1,0	1,0	8,5	3,5	7,6	0	1	1	appropriate
	Dispelling false myths (e.g., cervical headache)	d34_r05	134	8,19	9,0	1,3	6,0	3,0	9,0	8,0	8,0	9,0	9,0	9,0	1,0	1,0	8,5	3,5	7,6	0	1	1	appropriate
	Prevention through healthy lifestyles	d34_r06	134	8,23	9,0	1,2	5,0	4,0	9,0	8,0	8,0	9,0	9,0	9,0	1,0	1,0	8,5	3,5	7,6	0	1	1	appropriate
	Promotion of specialized migraine treatment centers nationwide	d34_r07	134	8,01	8,0	1,4	8,0	1,0	9,0	8,0	8,0	8,0	9,0	9,0	1,0	1,0	8,5	3,5	7,6	0	1	1	appropriate
1. For a girl of pubertal age who begins to suffer from recurrent attacks of headache with migraine characteristics, it is <u>always</u> necessary to request:	Hormone testing	Q2_d01_r01	126	4,4	4,5	2,7	8,0	1,0	9,0	1,8	2,0	4,5	6,0	7,0	5,3	4,0	4,0	1,0	3,9	1	0	10	uncertain
	Brain MRI	Q2_d01_r02	126	3,97	4,0	2,3	8,0	1,0	9,0	2,0	2,0	4,0	5,0	6,0	4,0	3,0	3,5	1,5	4,6	0	0	0	uncertain
	Gynecological examination	Q2_d01_r03	126	4,98	5,0	2,7	8,0	1,0	9,0	3,0	3,0	5,0	7,0	7,0	4,0	4,0	5,0	0,0	2,4	1	0	10	uncertain
	Psychological evaluation	Q2_d01_r04	126	4,05	4,0	2,3	8,0	1,0	9,0	2,0	2,0	4,0	5,0	6,0	4,0	3,0	3,5	1,5	4,6	0	0	0	uncertain
	General blood tests	Q2_d01_r05	126	5,95	7,0	2,5	8,0	1,0	9,0	4,8	5,0	7,0	8,0	8,0	3,3	3,0	6,5	1,5	4,6	0	1	1	appropriate
	Eye exam	Q2_d01_r06	126	4,74	5,0	2,9	8,0	1,0	9,0	1,0	2,0	5,0	7,0	7,3	6,3	5,0	4,5	0,5	3,1	1	0	10	uncertain
	Dental exam	Q2_d01_r07	126	3,52	3,0	2,5	8,0	1,0	9,0	1,0	1,0	3,0	5,0	5,0	4,0	4,0	3,0	2,0	5,4	0	-1	-1	inappropriate
2. In the case of menstrual-related headaches, the following should <u>always</u> be done:	Hormone testing	Q2_d02_r01	126	5,24	6,0	3,0	8,0	1,0	9,0	2,0	3,0	6,0	8,0	8,0	6,0	5,0	5,5	0,5	3,1	1	0	10	uncertain
	Brain MRI	Q2_d02_r02	126	3,1	3,0	1,9	7,0	1,0	8,0	1,0	1,0	3,0	4,0	5,0	4,0	3,0	2,5	2,5	6,1	0	-1	-1	inappropriate
	Pelvic ultrasound	Q2_d02_r03	126	4,76	5,0	2,9	8,0	1,0	9,0	2,0	2,0	5,0	7,0	8,0	6,0	5,0	4,5	0,5	3,1	1	0	10	uncertain
	Endocrinology consultation	Q2_d02_r04	126	4,59	5,0	2,8	8,0	1,0	9,0	2,0	2,0	5,0	6,0	7,0	5,0	4,0	4,0	1,0	3,9	1	0	10	uncertain
3. Treatment of menstrual migraine requires, depending on the case, hormone therapy (miniprophylaxis/contraceptive).		Q2_d03_r01	126	6,25	7,0	2,1	8,0	1,0	9,0	5,0	5,0	7,0	8,0	8,0	3,0	3,0	6,5	1,5	4,6	0	1	1	appropriate
4. Hormonal contraceptive therapy is the first line of treatment for women suffering from menstrual migraine and should be offered to all women affected by this condition.		Q2_d04_r01	126	4,51	4,0	2,6	8,0	1,0	9,0	2,0	3,0	4,0	6,0	7,0	5,0	3,0	4,5	0,5	3,1	0	0	0	uncertain
5. A woman suffering from migraine who is undergoing estrogen-progestin therapy requires gynecological follow-up.		Q2_d05_r01	126	7,4	8,0	1,8	8,0	1,0	9,0	7,0	7,0	8,0	9,0	9,0	2,0	2,0	8,0	3,0	6,9	0	1	1	appropriate
6. A woman with migraine undergoing estrogen-progestin therapy requires neurological follow-up.		Q2_d06_r01	126	6,95	7,0	2,0	8,0	1,0	9,0	6,0	6,0	7,0	8,0	9,0	3,0	2,0	7,0	2,0	5,4	0	1	1	appropriate
7. A woman suffering from migraine who is undergoing estrogen-progestin therapy always requires a cardiological examination.		Q2_d07_r01	126	3,96	4,0	2,4	8,0	1,0	9,0	2,0	2,0	4,0	5,0	6,0	4,0	3,0	3,5	1,5	4,6	0	0	0	uncertain
8. A woman with migraine who is trying to conceive needs gynecological follow-up.		Q2_d08_r01	123	7,41	8,0	2,0	8,0	1,0	9,0	7,0	7,0	8,0	9,0	9,0	2,0	2,0	8,0	3,0	6,9	0	1	1	appropriate
9. A woman with migraine who is trying to conceive requires neurological follow-up.		Q2_d09_r01	123	7,05	7,0	1,8	8,0	1,0	9,0	6,0	6,2	7,0	8,0	9,0	3,0	1,8	7,1	2,1	5,5	0	1	1	appropriate
10. A woman with migraine who is trying to conceive needs to plan her pregnancy in advance, even if she is not taking and does not need medication.		Q2_d10_r01	123	4,24	4,0	2,7	8,0	1,0	9,0	1,0	2,0	4,0	6,0	7,0	6,0	4,0	4,0	1,0	3,9	1	0	10	uncertain
11. Migraine during pregnancy is never a problem because it tends to resolve spontaneously.		Q2_d11_r01	123	2,98	2,0	2,1	8,0	1,0	9,0	1,0	1,0	2,0	3,8	4,0	3,0	2,8	2,4	2,6	6,3	0	-1	-1	inappropriate
12. A woman suffering from migraine during pregnancy only needs gynecological follow-up.		Q2_d12_r01	123	3,27	3,0	2,4	8,0	1,0	9,0	1,0	1,2	3,0	4,0	5,0	4,0	2,8	2,6	2,4	6,0	0	-1	-1	inappropriate
13. When should a woman with migraine who is breastfeeding stop breastfeeding in order to start drug prophylaxis?	If she has fewer than one attack per week controlled by acute therapy	Q2_d13_r01	123	2,09	1,0	1,6	7,0	1,0	8,0	1,0	1,0	1,0	2,0	3,0	2,0	1,0	1,5	3,5	7,6	0	-1	-1	inappropriate
	If she has fewer than one attack per week not controlled by acute therapy	Q2_d13_r02	123	3,48	3,0	2,1	8,0	1,0	9,0	1,0	2,0	3,0	5,0	5,0	4,0	3,0	3,5	1,5	4,6	0	-1	-1	inappropriate
	If she has more than one attack per week with insufficient response to non-pharmacological preventive therapies	Q2_d13_r03	123	6,49	7,0	2,3	8,0	1,0	9,0	5,0	6,0	7,0	8,0	8,0	3,0	2,0	7,0	2,0	5,4	0	1	1	appropriate
	Never	Q2_d13_r04	123	3,38	2,0	2,5	8,0	1,0	9,0	1,0	1,0	2,0	5,0	5,0	4,0	4,0	3,0	2,0	5,4	0	-1	-1	inappropriate
14. Hormone replacement therapy in perimenopausal women with migraine without aura:	It should be prescribed in asymptomatic patients for menopausal symptoms.	Q2_d14_r01	123	2,93	2,0	2,2	8,0	1,0	9,0	1,0	1,0	2,0	4,0	5,0	4,0	3,0	2,5	2,5	6,1	0	-1	-1	inappropriate
	It should be prescribed in symptomatic patients for menopausal symptoms.	Q2_d14_r02	123	7,02	7,0	1,6	8,0	1,0	9,0	6,0	7,0	7,0	8,0	8,0	2,0	1,0	7,5	2,5	6,1	0	1	1	appropriate
15. The first level of intervention for the prevention of cardiovascular and cerebrovascular disease in postmenopausal women with migraine without aura includes:	Prescription of specialist tests (hormonal and cardiovascular)	Q2_d15_r01	123	6,45	7,0	2,3	8,0	1,0	9,0	5,0	5,0	7,0	8,0	8,0	3,0	3,0	6,5	1,5	4,6	0	1	1	appropriate
	Hormone replacement therapy in women with symptoms of post-menopausal disorders	Q2_d15_r02	123	5,54	6,0	2,2	8,0	1,0	9,0	4,0	5,0	6,0	7,0	7,0	3,0	2,0	6,0	1,0	3,9	0	0	0	uncertain
